# Oncogenic Herpesvirus Utilizes Stress-Induced Cell Cycle Checkpoints for Efficient Lytic Replication

**DOI:** 10.1371/journal.ppat.1005424

**Published:** 2016-02-18

**Authors:** Giuseppe Balistreri, Johanna Viiliäinen, Mikko Turunen, Raquel Diaz, Lauri Lyly, Pirita Pekkonen, Juha Rantala, Krista Ojala, Grzegorz Sarek, Mari Teesalu, Oxana Denisova, Karita Peltonen, Ilkka Julkunen, Markku Varjosalo, Denis Kainov, Olli Kallioniemi, Marikki Laiho, Jussi Taipale, Sampsa Hautaniemi, Päivi M. Ojala

**Affiliations:** 1 Translational Cancer Research Program, Research Programs Unit, University of Helsinki, Helsinki, Finland; 2 Genome-Scale Biology Program, Research Programs Unit and Department of Pathology, Haartman Institute, University of Helsinki, Helsinki, Finland; 3 Genome-Scale Biology Program, Research Programs Unit, University of Helsinki, Biomedicum Helsinki, Helsinki, Finland; 4 VTT Medical Biotechnology, Turku, Finland; 5 Institute for Molecular Medicine (FIMM), University of Helsinki, Biomedicum Helsinki, Helsinki, Finland; 6 Centre for Drug Discovery, University of Helsinki, Helsinki, Finland; 7 Department of Virology, University of Turku and National Institute for Health and Welfare, Turku, Finland; 8 Department of Radiation Oncology, The Johns Hopkins University School of Medicine, Baltimore, Maryland, United States of America; 9 Foundation for the Finnish Cancer Institute, Helsinki, Finland; 10 Section of Virology, Division of Infectious Diseases, Department of Medicine, Imperial College London, London, United Kingdom; Wistar Institute, UNITED STATES

## Abstract

Kaposi’s sarcoma herpesvirus (KSHV) causes Kaposi’s sarcoma and certain lymphoproliferative malignancies. Latent infection is established in the majority of tumor cells, whereas lytic replication is reactivated in a small fraction of cells, which is important for both virus spread and disease progression. A siRNA screen for novel regulators of KSHV reactivation identified the E3 ubiquitin ligase MDM2 as a negative regulator of viral reactivation. Depletion of MDM2, a repressor of p53, favored efficient activation of the viral lytic transcription program and viral reactivation. During lytic replication cells activated a p53 response, accumulated DNA damage and arrested at G2-phase. Depletion of p21, a p53 target gene, restored cell cycle progression and thereby impaired the virus reactivation cascade delaying the onset of virus replication induced cytopathic effect. Herpesviruses are known to reactivate in response to different kinds of stress, and our study now highlights the molecular events in the stressed host cell that KSHV has evolved to utilize to ensure efficient viral lytic replication.

## Author Summary

Herpesviruses are known to wake up and reactivate in response to different kinds of stress. Our study now highlights the key molecular host cell events that KSHV has evolved to utilize for efficient viral lytic replication: the activation of p53 and upregulation of p21, which slows down the cell cycle, but promotes viral replication and transcription of viral lytic genes. Mutations in *TP53* gene are rarely found in KSHV-associated malignancies. Therefore, our work now provides a mechanistic explanation as to why the virus has evolved to retain p53.

## Introduction

Kaposi’s sarcoma-associated herpesvirus (KSHV) is a human tumor virus in the family of gamma2-herpesviruses. KSHV is the etiologic agent of Kaposi’s sarcoma (KS) and other KSHV-associated lymphoproliferative diseases such as primary effusion lymphoma (PEL) [1,2]. KSHV genome consists of linear double-stranded DNA (dsDNA), and like other herpesviruses, the virus displays two modes of infection in the infected cells, the latent and lytic replication phase. Upon entry into the host cell nucleus, the linear dsDNA genome circularizes forming a non-integrated viral episome that persists as multiple copies in the latently infected cells [3]. The latent infection (latency) provides an immunologically silent mode of persistence, whereas the lytic replication phase allows replication and production of new virions to be shed and transmitted to new cells and hosts.

The switch between the latency and lytic replication (virus reactivation) is a critical step in viral pathogenesis. Although the KSHV-associated tumors typically show low level of virus reactivation [4,5], epidemiological studies support the importance of lytic replication in the initiation and progression of KS [6–8]. Despite of active research, the regulation of viral reactivation is not completely understood. However, significant advances have been made in recent years, and the reported mechanisms of KSHV reactivation involve hypoxia [9–11], reactive oxygen species [12], inflammation [13–15], activation of cellular kinases [16–20] and epigenetic mechanisms [21–25]. KSHV reactivation can also be chemically induced e.g. with certain kinase agonists (TPA) and chemical inhibitors affecting histone acetylation (HDAC inhibitors) or DNA methylation (reviewed in [26]).

Recent studies by several groups have demonstrated that the intracellular viral genome has chromatin structures similar to that of the host chromosome (reviewed in [22]). The latent KSHV genome is epigenetically modified with methylation at CpG dinucleotides as well as mutually exclusive activating and repressive histone modifications [27–29]. The bivalent chromatin structure represents a poised state of repression during viral latency, which can be rapidly reversed once the lytic cycle is induced, and enables the virus to fine-tune its gene expression patterns in response to changes in virus infected cells. Further support for the importance of epigenetic regulation in the switch from latency to lytic replication was provided by the demonstration of the cohesin subunits as major repressors of KSHV lytic gene activation suggesting that cohesins could be a direct target of butyrate-mediated lytic induction [30]. Other recently identified epigenetic regulators of KSHV reactivation include the H3K27me3 histone methyltransferase of the Polycomb group proteins, EZH2 [28], HDAC class I and II [25], and the histone demethylase JMJD2A [31].

To discover novel mechanisms regulating KSHV reactivation we designed and performed a small interfering RNA (siRNA) screen using a library of siRNAs specific for human genes involved in epigenetic processes. In this screen we assessed which epigenetic enzymes help the virus to maintain latency. We identify MDM2, an E3 ubiquitin ligase, as a novel modulator whose depletion by siRNA accelerates KSHV reactivation. We also show that MDM2 down-regulation leads to subsequent activation of p53 and p21 as well as induction of a p21-dependent cell cycle arrest, which are required for the induction of efficient viral lytic replication cascade.

## Results

### siRNA screen identifies MDM2 as a novel regulator of KSHV lytic gene expression

To identify novel regulators of KSHV reactivation we performed a siRNA screen using a custom-made siRNA library targeting 615 human genes with Gene Ontology (GO) annotations related to epigenetics, chromatin remodeling/maintenance, and co-regulatory functions, and consisting of two independent siRNAs for each gene [32]. Before reverse transfection, the siRNAs, mixed with transfection reagent and components of the extracellular matrix, were spotted onto a microplate-sized array plate and analyzed by the cell-spot microarray technique (CSMA; [33]. For the screen, we used SLK cells stably infected with a recombinant KSHV (rKSHV.219) which during latency constitutively expresses green fluorescent protein (GFP) under the control of the cellular EF-1α promoter, and upon reactivation the red fluorescent protein (RFP) from the promoter of the viral early lytic gene PAN [34]. The workflow of the primary screen is depicted in S1A Fig (for details see S1 Methods).

Among the siRNAs that enhanced reactivation, one of the strongest and the most reproducible ones were the siRNAs against MDM2 (siMDM2). The depletion of MDM2 led to RFP levels that were three SDs above the mean RFP value for the screen (S1 Fig). In this study, we pursued validation and characterization of the role of MDM2 in KSHV lytic replication.

To validate the results of the screen, we tested two additional MDM2 siRNAs targeting different, non-overlapping sequences of the *MDM2* transcript. The siRNAs were pooled and transfected into iSLK.219 cells, a recently developed cell clone derived from SLK cells stably infected with rKSHV.219 [35]. These cells contain an exogenous copy of the viral gene RTA that can be induced by doxycycline. Addition of low doses of doxycycline results in efficient RTA synthesis (S2A Fig) that triggers virus reactivation in approximately 5–15% of cells at 24 hours post induction (hpi) (S2B and S2C Fig). This can be substantially augmented by the combination of doxycycline (Dox) with TPA (TPA/Dox) or NaB (NaB/Dox) which increase the fraction of RFP-positive cells to 80–90% within 24 hours of treatment (S2B and S2C Fig).

In the absence of Dox, treatments with TPA or NaB alone do not result in lytic reactivation (S2B and S2C Fig), thereby providing an invaluable control to monitor possible pleiotropic effects of the two compounds.

**Fig 1:**
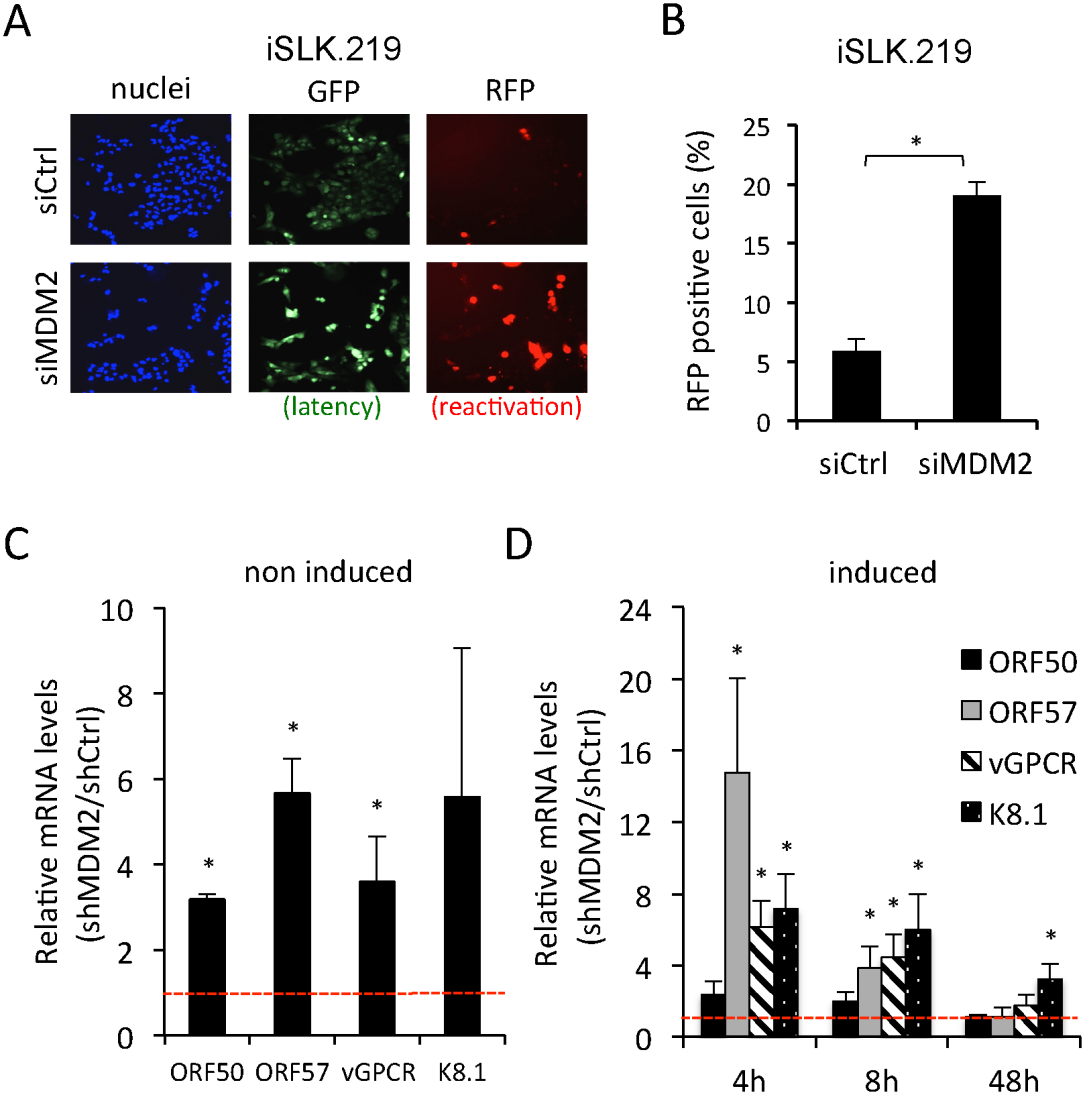
siRNA screen identifies MDM2 as a novel regulator of KSHV reactivation. (A) Fluorescence images of iSLK.219 cells treated with indicated siRNAs. Virus reactivation was induced 48h after siRNA transfection by a low-dose (0.05 μg/ml) doxycycline treatment and the extent of reactivation measured by detection of RFP positive cells using high-content fluorescence imaging and automated image analysis. Cells were visualized with Hoechst 33342 (Nuclei, blue), GFP (marker of latency, green) and RFP (marker of reactivation, red). (B) Quantification of the experiment shown in (A). The results are the average of three independent experiments. The error bars represent the standard error of the mean (SEM). * p<0.05. (C) qRT-PCR analysis of ORF50, ORF57, vGPCR and K8.1 transcripts from latent, DMSO-treated BC-3 cells transduced for 72 h with lentiviruses expressing nonspecific control (shCtrl) or MDM2 targeting shRNAs (shMDM2). The relative mRNA levels obtained from the shMDM2 treated cells were normalized to the corresponding values from shCtrl treated cells. (D) qRT-PCR analysis of ORF50, ORF57, vGPCR and K8.1 transcripts from BC-3 treated with shRNA as in (C) and reactivated with TPA for indicated times. Values normalized as in (C). The error bars in (C) and (D) represent the SEM of three independent experiments. * p<0.05

To assess the effect of MDM2 depletion in the iSLK.219 cells [35] we incubated the siRNA transfected cells for 72 hours (h), and treated the cells by suboptimal doses of doxycycline that alone produce no or very low level of RFP induction. After fixation, cells were imaged and analyzed for RFP expression using automated high-content fluorescence microscopy. The depletion efficiency of the MDM2 siRNAs was 62% as monitored by quantitative real time PCR (qRT-PCR). As shown in Fig 1A and 1B, and confirming the results of the screen, depletion of MDM2 led to a 3-fold increase in the number of RFP—positive cells compared to the control siRNA transfected cells.

To further validate the results of the screen in cells that are naturally infected by KSHV, we silenced MDM2 expression in BC-3 cells, a patient-derived primary effusion lymphoma (PEL) cell line[36]. BC-3 cells were transduced with lentiviruses expressing shMDM2, or shCtrl, and 72 h later virus reactivation was induced by TPA treatment. We first assessed whether the depletion of MDM2 induced spontaneous reactivation by analyzing the mRNA levels of the lytic genes ORF50 (encoding for RTA; immediate early), ORF57 (encoding for MTA; delayed early), vGPCR (intermediate) and K8.1 (late) in non-induced BC-3 cells. Whole cell extracts were collected at indicated times and viral lytic transcripts were analyzed by qRT-PCR (Fig 1C and 1D). Depletion of MDM2 induced the expression of the viral transcripts by 3.2 to 5.6 fold compared to controls (Fig 1C) indicating spontaneous viral reactivation. Similarly, during TPA-induction MDM2 depletion led to a rapid increase of all tested viral lytic transcripts that ranged from 2.5 (ORF50) up to 15 fold (ORF57) at 4 hpi (Fig 1D). Interestingly, over time, the magnitude of the up-regulation gradually decreased, reaching control levels at 48 hpi. Thus, silencing of MDM2 in PEL cells led to spontaneous reactivation and accelerated the kinetics of lytic gene expression, without altering their maximal levels during the time course studied.

### Viral reactivation leads to a p53 response in PEL cells

MDM2 is the major regulator of the tumor suppressor p53 and targets p53 to proteasomal degradation through its ubiquitin E3 ligase activity [37]. While p53 is inactivated during KSHV latency [38–45] its potential role during the lytic phase of the virus replication cycle has not been investigated.

The possibility that the transcription factor p53 could be required for efficient viral lytic gene expression prompted us to conduct a comprehensive, unbiased analysis of the p53 chromatin binding sites during the reactivation of BC-3 cells. To this end, we carried out chromatin immunoprecipitation using antibodies against p53, followed by genome-wide deep sequencing (ChIP-seq) analysis of the associated DNA. Non-specific IgG antibody was used as a negative control. BC-3 cells were subjected to ChIP-seq after TPA induction for 0 or 24 h. As a positive control for detection of the canonical p53 targets, non-induced BC-3 cells were treated with Nutlin-3 (here referred to as Nutlin), a small molecule that binds and inhibits MDM2, stabilizing p53 and inducing a potent p53 response.

**Fig 2:**
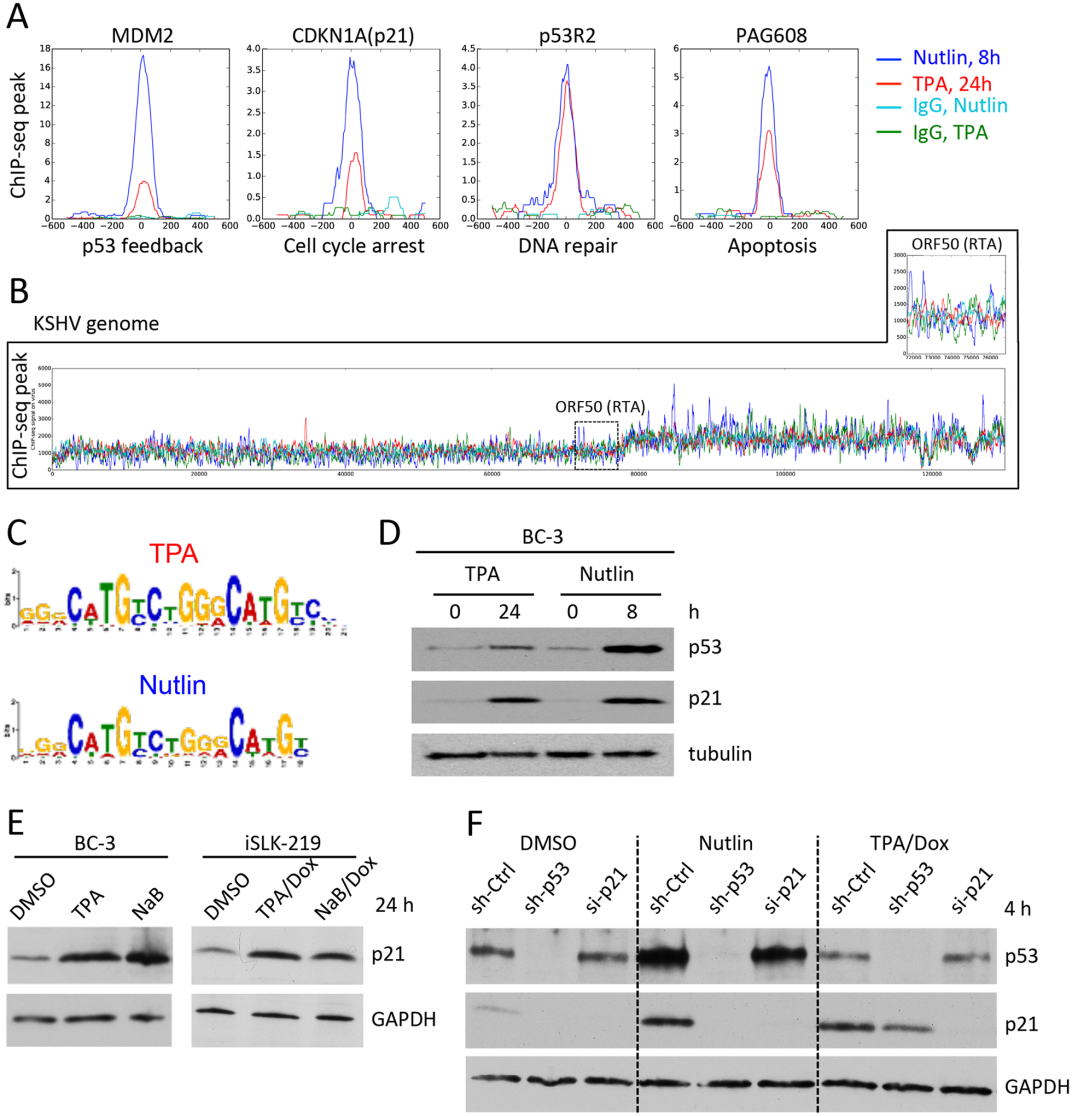
Viral reactivation leads to a p53 response in PEL cells. (A) BC-3 cells treated with TPA (red line) or Nutlin (blue line) for indicated times were processed for ChIP-seq analysis using an antibody against p53. Each chart represents the sequencing signal (ChIP-seq peak) obtained after immunoprecipitation with the p53 (blue and red lines) or nonspecific IgG antibodies (light blue and green lines) for the indicated cellular genes. The known function of each gene is indicated at the bottom of each panel. (B) The sequencing signal of BC-3 cells treated as in (A) is shown for the whole KSHV genome. The inset is an enlarged view of the DNA region coding for the RTA gene (black dashed box). (C) Using the MEME software, the same *consensus* binding-motif of p53 was identified *de novo* from the ChIP-seq results of BC-3 cells treated with Nutlin (8 h) or TPA (24 h). (D) BC-3 cells were treated with TPA for or Nutlin for indicated times and processed for WB using antibodies against p53, p21 and tubulin. (E) BC-3 or iSLK.219 cells treated with vehicle (DMSO) or indicated inducers for 24 h were processed for WB using antibodies against p21 and GAPDH. (F) iSLK.219 cells were treated with indicated sh- or siRNAs for 48 h and then incubated with DMSO, Nutlin or TPA/Dox for 4 h before WB analysis using antibodies against p53, p21 and GAPDH.

Compared to the DMSO-treated cells, the TPA treatment induced a global activation of p53 that could be visualized by averaging the sequencing signal over the whole cellular genome (S3A Fig). Specific sequencing signal was found at regions preceding known p53-target genes, such as MDM2, CDKN1A (p21Cip1), P53R2 and PAG608 (Fig 2A). For some genes, the sequencing peak obtained from TPA-treated cells was comparable to that of Nutlin treated cells (Fig 1A, p53R2; S3B Fig, red arrow heads). Pathway analysis indicated that the genes close to the 300 most significant p53 binding sites (p<0.01) were involved in Cell cycle arrest, Apoptosis, DNA repair and p53 regulation (i.e. MDM2) (S3C Fig). 94 of the 99 peaks with the lowest p-values from the TPA sample overlapped with the peaks identified from Nutlin-treated cells (S3D Fig), confirming that in BC-3 cells TPA treatment induced a *bona fide* p53 response.

Notably, the ChIP-seq analysis of TPA- or Nutlin-treated cells did not produce statistically significant p53 binding events on the viral genome. The sequencing signal from TPA- or Nutlin- treated cells (Fig 2B red and blue lines, respectively) was comparable to the background noise obtained with the nonspecific IgG controls (Fig 2B, light blue and green lines, respectively) despite the high copy number of viral genomes per cell in BC-3 and PEL cells in general [46]. The absence of p53-binding sites on the viral genome prompted us to perform a quality control to confirm that the p53 binding sites identified in the cellular DNA corresponded to the expected consensus sequences [47]. A *de novo* identification of the p53 DNA-binding sequence returned its known consensus sequence (Fig 2C) [47], for both TPA and Nutlin treated cells.

Thus, if p53 was involved in virus reactivation, this effect occurred through the activation of cellular rather the viral genes.

Of the different pathways induced by p53, we investigated the role of cell cycle arrest, a phenomenon known to occur during the lytic phase of herpesviruses and other DNA viruses [48]. Of particular interest was p21Cip1 (p21), a strong inhibitor of cell cycle progression [49], which was also identified in our ChIP-seq analysis of TPA induced cells (Fig 2A and S3B and S3C Fig).

To validate the results of the ChIPseq experiment, we determined the level of p53 and its target p21 by immunoblotting of cell extracts collected at indicated times after reactivation with TPA or Nutlin treatment (Fig 2D). Compared to the DMSO controls (0 h), induction with TPA caused a small increase in the levels of p53 and a strong induction of p21 (Fig 2D).

The up-regulation of p21 was fast and could be detected already 4 h after TPA treatment in BC-3 cells (S4E Fig). The p21 levels also increased in iSLK.219 cells treated with TPA/Dox or NaB/Dox (Fig 2E). To test if this effect was due to viral lytic gene expression, we compared by WB the levels of p21 in iSLK.219 cells treated with DMSO (non induced), Dox, TPA or TPA/Dox (S3E Fig). Viral lytic gene expression was monitored with antibodies against MTA (ORF57, early lytic gene). In iSLK.219 cells, TPA treatment is not sufficient to trigger lytic reactivation (see also S2B and S2C Fig). However, the levels of p21 in cells treated with TPA alone for 4h were comparable to those in cells treated with TPA/Dox that results in lytic reactivation (S3E Fig). No increase in p21 levels was observed after 4h treatment with Dox that leads to the synthesis of RTA (ORF50) from a plasmid stably maintained in the iSLK.219 cells. Four hours after Dox treatment the synthesis of lytic genes downstream of RTA is still not detectable by WB. Thus, the fast increase in p21 levels was not due to viral lytic gene expression but appeared to be an intrinsic effect of the TPA treatment. To confirm this, we incubated non-infected SLK cells with similar doses of TPA/Dox or NaB/Dox and monitored by WB the levels of p21 over time. Nutlin treatment was used as a positive control. Although with slower kinetics, TPA and NaB induced an increase of p21 levels by 12 h (S3F Fig). These results are consistent with numerous earlier reports obtained in other cell types [50–53].

To test whether the induction of p21 was p53-dependent we depleted p53 in iSLK.219 cells and then monitored the levels of p21 after treatment with DMSO, Nutlin or TPA/Dox. At 48 h after transduction with lentiviruses expressing shRNA against p53 (sh-p53) or nonspecific non-targeting controls (sh-Ctrl), iSLK.219 were treated with indicated compounds for 4 h and the levels of p53 and p21 were monitored by WB of whole cell extracts (Fig 2F). The specificity of the p21 antibody was confirmed by including iSLK.219 cells treated with siRNAs against p21 (si-p21). Compared with DMSO controls, treatment with Nutlin increased the levels of p53 and p21. As in our previous results in BC-3 cells (Fig 2D), the levels of p21 in Nutlin and TPA/Dox treated cells were comparable despite the large difference in the levels of p53 (Fig 2F, Nutlin and TPA/Dox, sh-Ctrl lanes). Depletion of p53 in DMSO- and Nutlin-treated cells was very efficient and abrogated the induction of p21 (Fig 2F, DMSO and Nutlin). Upon TPA/Dox treatment, however, p21 was induced even in the absence of detectable p53 (Fig 2F, TPA/Dox, sh-p53 lanes), indicating that upon TPA/Dox induction the levels of p21 are regulated by both p53-dependent and -independent mechanisms.

### p53 and p21 are required for efficient virus lytic gene expression

**Fig 3:**
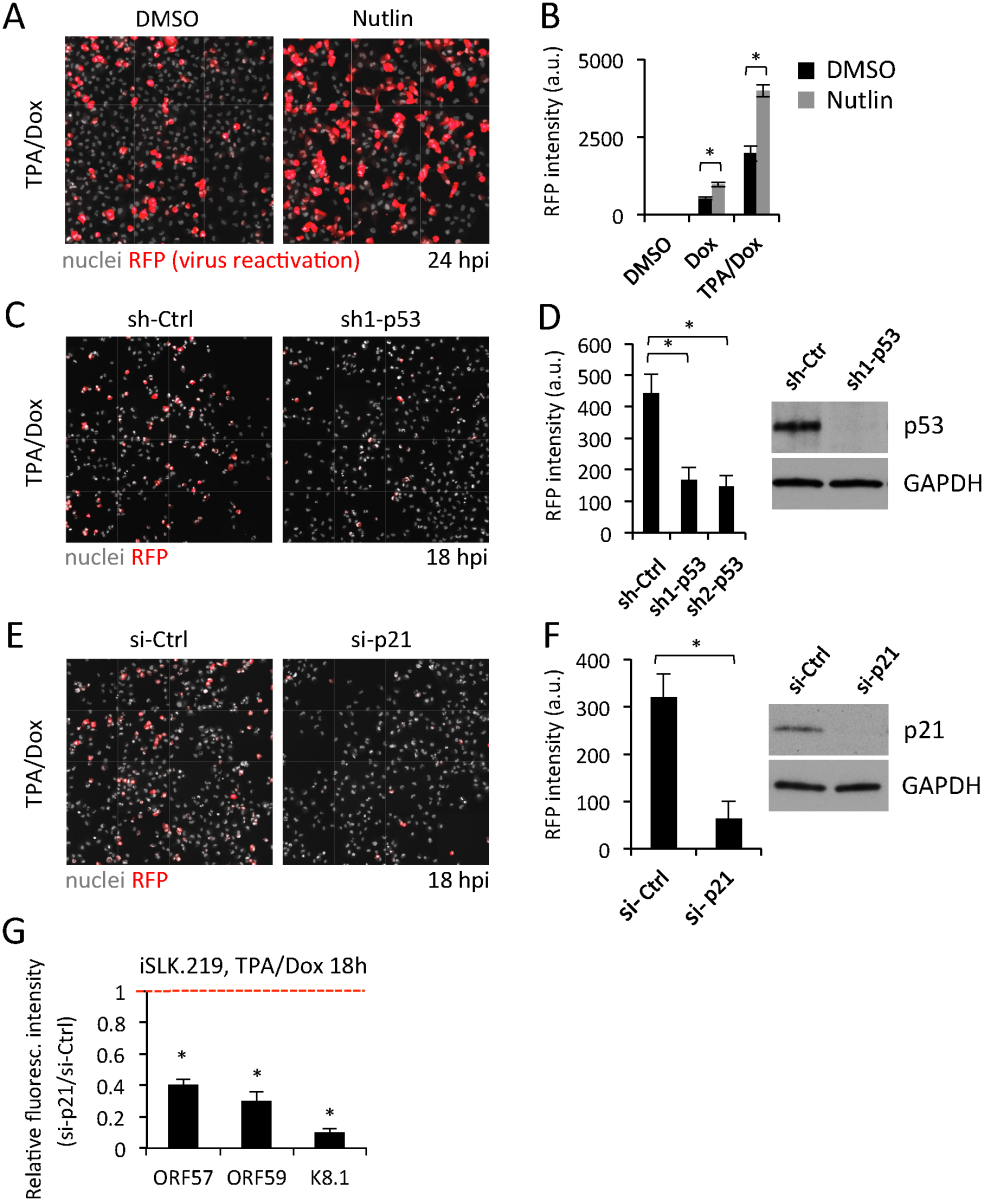
p53 and p21 are required for efficient virus lytic gene expression. (A) Fluorescence images of lytic gene expression (RFP, red) in iSLK.219 co-treated with TPA/Dox and DMSO or Nutlin for 24 h. Nuclei are stained with Hoechst (grey). (B) Quantification of the median RFP intensity from the experiment shown in (A). Values represent the mean of three independent experiments. Error bars indicate the SD. * p<0.05. (C) Fluorescence images of RFP (Red) expression in iSLK.219 cells transduced for 48 h with lentiviruses expressing shRNA against p53 (sh1-p53) or non-targeting controls (sh-Ctrl), and reactivated with TPA/Dox for 18 h. Nuclei are stained with Hoechst (grey). (D) Quantification of the median RFP intensity from the experiment shown in (C). A second, independent shRNA against p53 (sh2-p53) was also included. The values are the mean of three independent repetitions. Error bars represent the SD. The efficiency of p53 depletion was monitored in parallel experiments by WB (right panel). * p<0.05. (E) Images of RFP (Red) expression in iSLK.219 cells transfected with siRNAs against p21 (si-p21) or non-targeting controls (si-Ctrl), and treated with TPA/Dox for 18 h. Nuclei are stained with Hoechst (grey). (F) Quantification of the median RFP intensity from cells treated as in (E). The values are the mean of three independent repetitions. Error bars represent the SD. Confirmation of the p21 depletion by WB is shown in the right panel. * p<0.05. (G) Quantification of lytic gene expression in iSLK.219 cells treated as in (E) and processed for immunofluorescence and image analysis. Values are normalized to the respective nonspecific controls (si-Ctrl) set to one (dotted red line). Each value represents the mean of three independent experiments and associated SD. * p<0.05

To test if an increase in p53/p21 levels would favor virus lytic gene expression we induced iSLK.219 cells with Dox or TPA/Dox in the presence of Nutlin for 24 h, and measured the levels of RFP (virus reactivation) by automated microscopy. In each experiment, we monitored the stabilization of p53 by Nutlin using immunofluorescence (S4A Fig). In parallel experiments using non-induced iSLK.219 cells, the decrease in the number of cells after Nutlin treatment was used to measure the proliferation arrest in response to p53 stabilization (S4B Fig). Consistent with the results obtained by depletion of MDM2, inhibition of MDM2 by Nutlin led to a two-fold increase in virus reactivation (Fig 3A and 3B).

Conversely, depletion of p53 using RNAi decreased RFP expression induced by TPA/Dox in iSLK.219 cells (Fig 3C and 3D). In these experiments the silencing of p53 was monitored by WB analysis (Fig 3D, insert). Similar results were obtained in TPA and NaB treated BC-3 cells, where the levels of lytic gene expression were monitored by qRT-PCR and WB (S4C–S4E Fig).

We then addressed the relevance of p21 in mediating the lytic reactivation. Similarly to the depletion of p53, siRNA-mediated depletion of p21 in TPA/Dox induced iSLK.219 cells decreased fluorescence intensity of RFP (Fig 3E and 3F) and other viral lytic genes (Fig 3G). To address the role of p21 for efficient viral lytic replication in a biologically relevant KSHV-infection system, we chose to use two PEL cell lines, TPA-treated BC-3 and TREx BCBL1-Rta (here referred to as BCBL1_RTA_) cells that can be induced to lytic replication through doxycycline-inducible expression of RTA [54]. We stably depleted p21 in both cell lines by transducing them with lentiviruses expressing a control unspecific shRNA (sh-Ctrl) or two different sh-p21 constructs (sh1-p21, sh2-p21) followed by puromycin selection. Twelve days after selection, both BC-3 and BCBL1_RTA_ cell lines expressing the sh-p21 constructs had growth kinetics undistinguishable from their respective sh-Ctrl transduced cells (S5A and S5B Fig).

**Fig 4:**
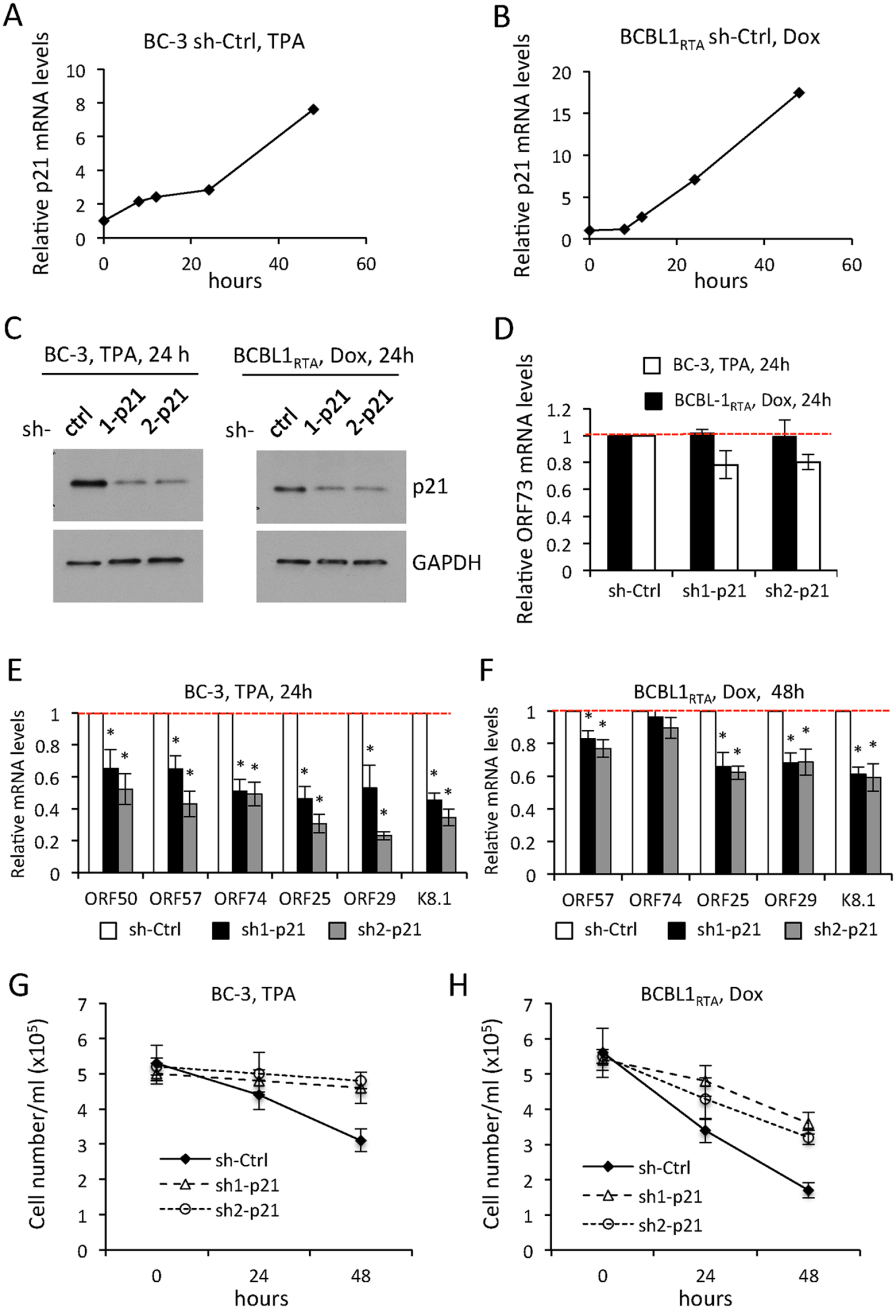
p21 depletion impairs lytic gene expression in PEL cells. (A-B) Quantification of p21 mRNA levels by qRT-PCR during a time course of TPA or doxyxyclin induced reactivation in the BC-3 and BCBL1_RTA_ PEL cells, respectively, stably expressing non specific sh-Ctrl. (C) WB analysis of p21 levels in whole cell extracts from PEL cells stably expressing indicated shRNAs and induced to reactivation with TPA (BC-3) or Dox (BCBL1_RTA_) for 24 h. GAPDH was used as a loading control. (D) Quantification of ORF73 (LANA) mRNA levels by qRT-PCR in PEL treated as in (C). Values represent the average and SEM of three replicates, and are normalized to the shCtrl set to one (red dashed line). (E-F) Quantification of transcription of the indicated lytic genes in PEL cells stably expressing sh-Scr, sh1-p21 or sh2-p21 and induced by TPA for 24h (BC-3, E) or Dox for 48h (BCBL1_RTA_, F). The values represent the mean and SDM of three independent experiments, and are normalized to sh-Ctrl set to one (red dashed line). *p<0.05. (G-H) Quantification of cell number to monitor virus replication induced cytopathic effect in PEL cells expressing indicated shRNAs and induced to lytic reactivation by TPA (BC-3, G) or Dox (BCBL1_RTA_, H). The values represent the mean and SDM of three independent experiment.

As shown for BC-3 and iSLK.219 cells (Fig 2D and 2E), the levels of p21 mRNA also increased in these new cell lines upon TPA (Fig 4A, BC-3 sh-Ctrl) or doxycycline (Fig 4B, BCBL1_RTA_ shCtrl) treatments.

The silencing efficiencies of p21 were monitored by qRT-PCR (S5C and S5D Fig) and WB (Fig 4C). Stable depletions of p21 had little or no effect on the expression levels of the latent gene ORF73 (Fig 4D) at 24h after TPA or Dox induction in BC-3 or BCBL1_RTA_ cells, respectively (Fig 4D). However, the expression levels of early, intermediate and late lytic genes were significantly decreased at 24 h (Fig 4E) or 48 h (Fig 4F) after induction of reactivation in BC-3 and BCBL1_RTA_, respectively. Of the two cell lines used, BC-3 cells were more sensitive to the depletion of p21 than the respective BCBL1_RTA_ cells (compare Fig 4E and 4F).

Thus the expression of p53 and p21 favored efficient viral lytic gene expression in both TPA-treated and RTA-overexpressing naturally KSHV-infected cells. Moreover, the decreased lytic gene expression in the p21-depleted cells resulted in a reproducible and significant delay in the onset of cytopathic effect (cell lysis), a known consequence of efficient virus replication (Fig 4G and 4H). Interestingly, despite the significant reduction of viral lytic gene expression, we saw only a modest decrease (up to approximately 30%) in the amount of infectious viruses released in the supernatant of p21-depleted BCBL1_RTA_ cells compared with their respective shCtrl expressing cells after reactivation by doxycycline for 24h (S5E and S5F Fig).

### KSHV-infected cells arrest at G2 phase during lytic replication

**Fig 5:**
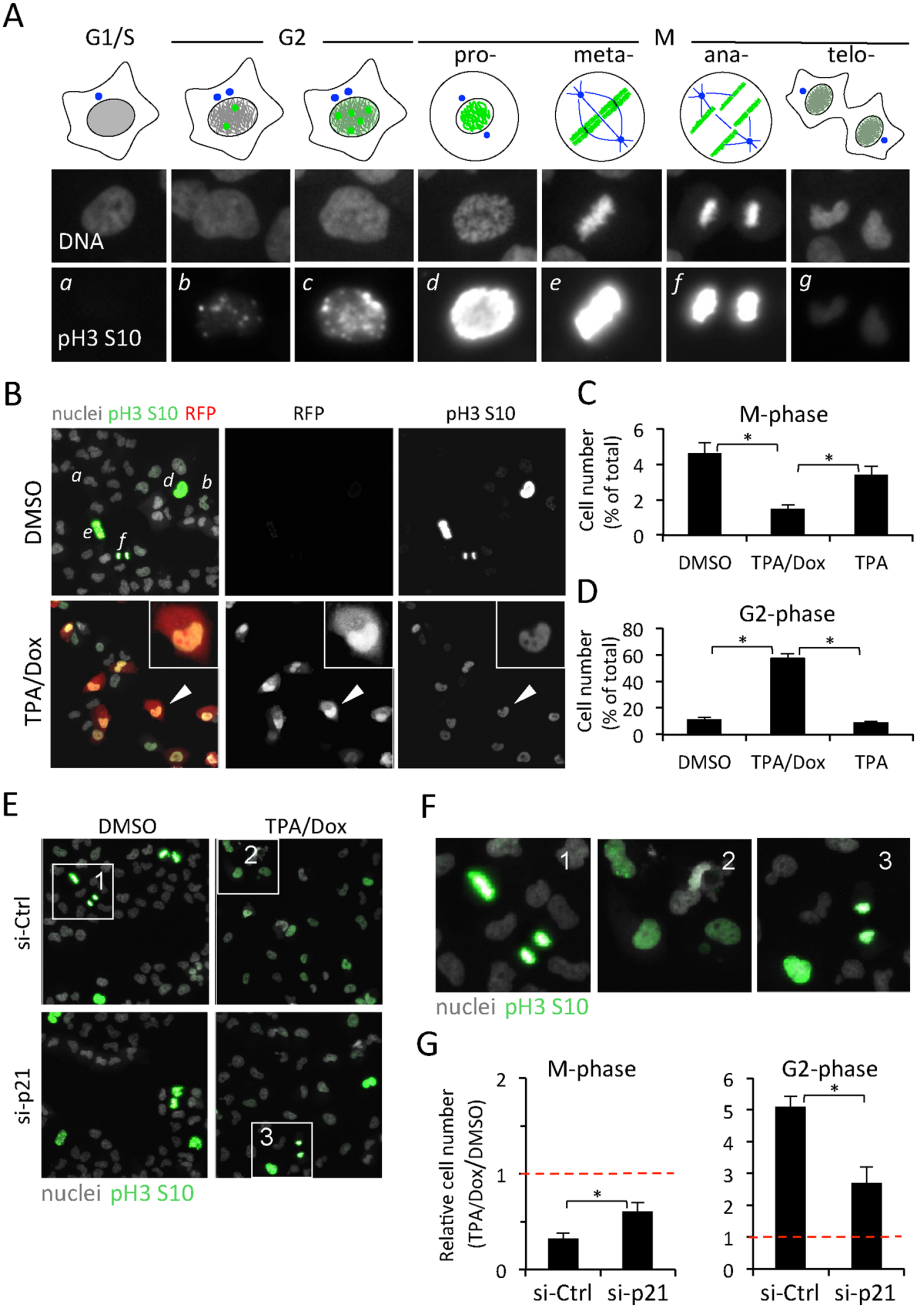
KSHV reactivation in iSLK.219 cells induces a G2 cell-cycle arrest. (A) Development of a high-content, image-based cell-cycle progression analysis based on the levels of phosphorylation of the histone H3 on serine-10 (pH3-S10). Automated image analysis was used to quantify the fluorescence intensity of each nucleus after immunofluorescence staining of pH3-S10, and to assign the corresponding cell to a specific stage of the cell cycle (*a* for G1/S, *b-e* for G2 or *f-g* for M). (B) Representative images of iSLK cells treated with DMSO or TPA/Dox for 20h and analyzed for cell cycle progression by pH3-S10 staining (green) and reactivation by RFP (red). Italics letters indicate different stages of the cell cycle (compare to (A)). The inset shows a magnification of the cell pointed by the white arrow head. (C-D) Quantification of the number of cells in M- (C) or G2-phase (D) in iSLK.219 cells treated with DMSO, TPA/Dox or TPAas. Values are the mean and SD of three independent experiments. The total number of cells in each sample was set as 100%. In each repetition, more than 1800 cells were analyzed. * p<0.05. (E) Images of pH3-S10 in iSLK.219 cells transfected with siRNAs against p21 (si-p21) or non-targeting controls (si-Ctrl), and reactivated with TPA/Dox for 20 hours. (F) Higher magnification images of cells in the respective white-box areas in panel (E). (G) Quantifications of the number of cells in M- or G2-phase in iSLK.219 cells treated as in (E). Values are normalized to the respective non-induced (DMSO) controls set to one (dotted red line). Error bars represent the SD of three independent experiments. * p<0.05.

p21 can arrest the cell cycle at G1/S or G2 phase by inhibiting the cyclin/Cdk complexes [49]. To assess how these properties of p21 contribute to the KSHV lytic reactivation, we developed an automated, image-based assay in which we used iSLK.219 cells and monitored viral reactivation and cell cycle progression at single-cell level. The expression of RFP served as a marker of virus reactivation, while cell cycle progression was monitored by immunofluorescence analysis using antibodies against histone H3 phosphorylated on serine 10 (pH3-S10). In dividing cells that enter the G2 phase, this antibody gives a punctate signal that labels the sites of chromatin condensation. As cells progress towards prometaphase, chromatin condensation continues and the levels of pH3-S10 increase proportionally, reaching a maximum at metaphase and anaphase, when the cellular DNA is tightly packed into chromosomes [55]. At the end of mitosis, histone H3 is rapidly dephosphorylated, and is undetectable in G1 and S phases. These stages could be easily distinguished in the iSLK.219 cells with automated fluorescence microscopy (Fig 5A). Using the fluorescence-intensity of the pH3-S10 staining and automated image analysis, cells were assigned to G1/S (no pH3-S10 signal, panel 'a'), G2 (weak and punctate pH3-S10 signal, panels 'b' and 'c', respectively) or M phase (bright pH3-S10 signal; panels 'd-f') (Fig 4A and S6A Fig). To further demonstrate that cells displaying pH3-S10 fluorescence are indeed in G2 or M-phase, we co-stained non-induced iSLK.219 cells with cyclin B1, another cellular marker of G2 and M-phase. As expected, the two markers were perfectly correlated (S6B Fig). Cells in G2 displayed a weak and punctate pH3-S10 signal and cytoplasmic cyclin B1, while cells in M-phase had bright and diffused pH3 S10 fluorescence and nuclear cyclin B1 (S6B Fig).

To test the accuracy of our image analysis method, we used as controls Nutlin, that arrests cells in G1/S, and Etoposide, an inhibitor of cellular topoisomerase-II that causes DNA damage and a G2/M cell cycle arrest (S6C and S6D Fig). Compared to DMSO controls, both Nutlin and Etoposide treatments strongly decreased the number of M phase cells (S6D Fig). Consistent with a robust G1/S arrest, Nutlin also decreased the number of cells in G2, while Etoposide increased the fraction of cells in G2 by more than six fold compared to DMSO treated samples (S6C and S6D Fig). Treatment with TPA/Dox led to an increase in the fraction of cells in G2, suggesting that during reactivation iSLK.219 cells arrest in G2 (S6C and S6D Fig, TPA/Dox). In parallel experiments, we excluded cross-talk between the RFP and Alexa488 fluorescence signal used to detect pH3 S10 (S7A Fig). Using similar drug treatments, the image analysis based cell cycle measurements were confirmed by traditional FACS analysis using propidium iodide to stain DNA (S8 Fig). Also in this case, the induction of iSLK.219 cells with TPA/Dox induced an increase in the fraction of S- and G2/M-phase cells, similar to the effect of etoposide treatments (S8B–S8G Fig). In these experiments the settings of the FACS detection were adjusted such that the RFP fluorescence did not contribute to the detection of PI (S8D Fig). Although very sensitive and in agreement with the results of the image analysis, the PI FACS analysis method could not distinguish between cells in G2- or in M-phase.

Based on the pH3 S10 imaging method, about 85% of non-induced iSLK.219 cells were at G1/S phase, 10% at G2 and 5% at M phase (Fig 5B and 5C, DMSO). At 24 h after TPA/Dox treatment, 62% of cells expressed RFP (compare with S2C and S2D Fig). The number of cells in M phase decreased from 4.6% to 1.5%, and the majority of reactivated cells (77% of all cells and 86% of RPF positive cells) displayed the weak pH3-S10 signal indicating an arrest in G2 phase (Fig 5B and 5C, TPA/Dox). To confirm that reactivated cells arrest in G2 we repeated the experiment using antibodies against cyclin B1. Indeed, compared to DMSO controls (S7B Fig), the vast majority of RFP positive cells also contained cyclin B1 (S7C Fig).

In the absence of Dox, TPA treatment did not induce G2 arrest, but only slightly decreased the number of M phase cells (Fig 5C and 5D, TPA). Thus viral lytic gene expression coincided with the G2 arrest.

To test whether depletion of p21 affected the G2 arrest observed during KSHV reactivation we silenced p21 for 48 h and subsequently monitored the levels of pH3-S10 in iSLK.219 cells treated with TPA/Dox for 24 h (Fig 5E–5G). As in previous experiments, induction with TPA/Dox induced a G2 arrest in cells treated with si-Ctrl (Fig 5E–5G, si-Ctrl). However, depletion of p21 in the TPA/Dox treated cells resulted in a significant decrease in the fraction of cells in G2 and a correspondent increase in the number of mitotic profiles to a level approaching the non-induced cells (Fig 5E–5G).

### During lytic reactivation, iSLK.219 cells bypass the G1 checkpoint and accumulate DNA damage

**Fig 6:**
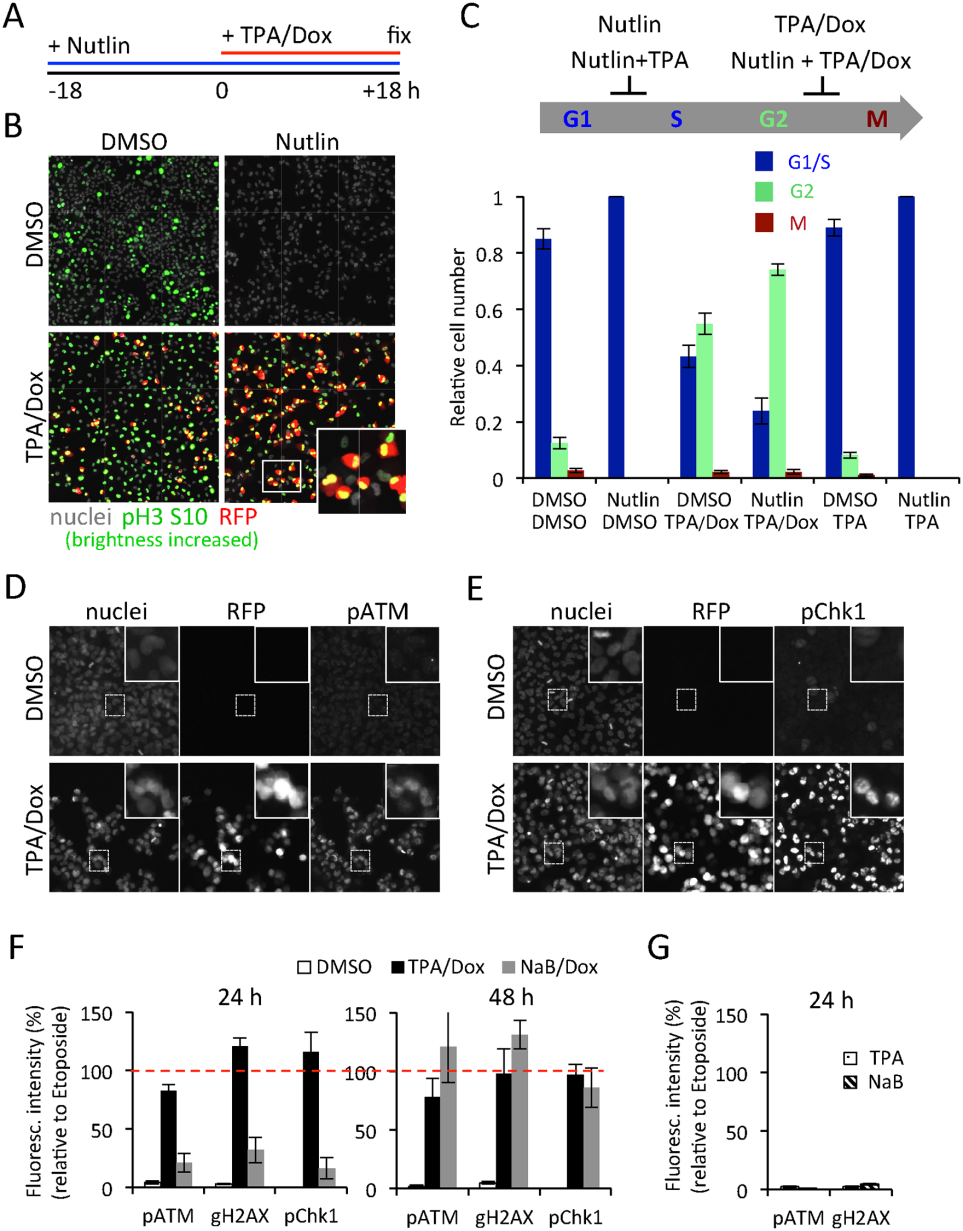
During lytic reactivation iSLK.219 cells 'by-pass' the G1-checkpoint and accumulate high levels of DNA damage. (A) Schematic representation of the Nutlin pretreatment experiment. iSLK.219 cells were treated with Nutlin 18h before reactivation with TPA/Dox. Cells were fixed 18h later and processed for immunofluorescence and high-content imaging. (B) Fluorescence images of iSLK.219 cells treated as in (A) and processed for immunofluorescence using antibodies against pH3 S10 (green). Lytic reactivation was monitored by RFP (red) expression, nuclei were stained with Hoechst. (C) Quantification of cell cycle progression by image analysis from the experiment shown in (B). In each condition, the values are normalized to the total number of cells (set as one). The cell cycle arrest induced by the different treatments is summarized in the scheme above the chart. (D-E) Representative fluorescence images of iSLK.219 cells reactivated with TPA/Dox for 24 h and processed for immunofluorescence staining of two markers of DNA damage response, phosphorylated ATM (pATM, D) and phosphorylated Chk1 (pChk1, E). The insets show higher magnifications of cells from the respective white-boxed areas. Nuclei are stained with Hoechst and virus reactivation monitored by RFP expression. (F) Quantification of the median fluorescence intensity of indicated markers of DNA damage in cells treated as indicated for 24 h or 48 h and processed for immunofluorescence analysis. For each marker, values were normalized to the median fluorescence intensities obtained from iSLK.219 cells treated with Etoposide (6.25 μM) for the same times (100%, red-dashed line). Error bars represent the SD of three independent experiments. (G) The fluorescence intensity of indicated DNA damage response was quantified as in (F) in cells treated with TPA or NaB for 24 h.

Upon lytic reactivation, the levels of p21 rise within the first 4 h, and increasing its levels with Nutlin did result in a complete G1/S arrest in latently infected cells. Why didn't reactivated cells then arrest in G1/S? If a mechanism existed to inactivate the G1/S checkpoint during the lytic phase, then reactivated cells would move on to the S-phase and reach the G2. To test this possibility, we pre-treated non-induced iSLK.219 cells with Nutlin for 18 h to induce a complete G1/S arrest, and then triggered virus reactivation by TPA/Dox in the presence of Nutlin. DMSO served as a control (Fig 6A). Cells were then fixed 18 h after reactivation and, the cell cycle progression was monitored by image analysis of pH3-S10 as described before.

As expected, after Nutlin treatment the non-induced cells arrested in G1/S (Fig 6B and 6C, Nutlin/DMSO). Again, TPA/Dox treatment arrested most of the cells in G2 (Fig 6B and 6C, DMSO/TPA/Dox). Strikingly, despite the Nutlin pre-treatment, the addition of TPA/Dox led to an increase in the fraction of cells in G2 while those in G1/S phase decreased (Fig 6B and 6C, Nutlin/TPA/Dox). This was not observed in the non-infected SLK cells used as a control (S9 Fig). In the absence of Dox, TPA treatment alone does not reactivate the virus in iSLK.219 cells and did not allow the cells to overcome the Nutlin-induced G1/S arrest (Fig 6C, Nutlin/TPA). Thus, during virus reactivation the G1/S checkpoint is inactivated while the G2/M checkpoint remains active.

The G2 arrest in lytic cells suggested activation of a DNA damage response (DDR). We and others have previously reported DNA damage induced by KSHV [56]. We therefore next assessed whether TPA/Dox or NaB/Dox activated DDR in iSLK.219 cells. DDR was monitored by immunofluorescence using antibodies against the phosphorylated forms of the Ataxia telangiectasia mutated (pATM, S1981) kinase, histone H2AX (γ-H2AX), and checkpoint kinase 1 (pChk1, S317) proteins (Fig 6D–6F). Non-induced iSLK.219 cells treated with Etoposide (10μM) served as positive controls (Fig 6F, dashed red line). Based on automated quantitative immunofluorescence analyses TPA/Dox-induced RFP-positive cells expressed robustly all DDR markers to a similar degree as the Etoposide-treated cells (Fig 6D–6F). The kinetics of DDR response was slower in the NaB/Dox treated cells but became comparable to those of TPA/Dox treated cells at 48 hpi (Fig 6F). Treatments with TPA or NaB in the absence of Dox, which is not sufficient to induce virus reactivation in this cell line, did not result in detectable DDR at 24 h (Fig 6G). Thus, reactivated cells accumulated DNA damage and activated DDR. Similar to the G2 arrest, the DDR was not due to pleiotropic effects of the TPA of NaB treatments but required viral lytic gene expression.

### Inhibitors of the DNA damage response are not effective to prevent cell cycle arrest and induce cell death in reactivated cells

When cells accumulate DNA damage, cell cycle arrest is necessary to allow DNA repair and prevent the propagation of damaged DNA to dividing cells, which would otherwise induce cell death during the M phase. Given the extent of DDR accumulated during the lytic phase, we hypothesized that in iSLK.219 the G2 arrest could prevent premature cell death.

The G2 arrest is enforced by the effectors of DDR, such as the kinases Chk1 and 2. We therefore tested the ability of different inhibitors of DDR (S10A Fig) to impair the G2 arrest and cause death in cells reactivated by TPA/Dox (lytic reactivation). The assay was optimized using increasing concentrations of Etoposide and caffeine, a broad inhibitor of ATM, ATR and other cellular kinases. The extent of DNA damage and cell cycle progression were monitored by immunofluorescence using antibodies against γH2AX and pH3-S10, respectively. Cells were treated with caffeine 1 h prior to addition of Etoposide, fixed 48 h later and processed for image analysis. Through inhibition of the upstream signals of the DDR (ATM/ATR), caffeine was very effective in inhibiting the Etoposide-induced G2 arrest and induced ‘mitotic catastrophe’ (S10B and S10C Fig; compare insets 1,2 and 3).

Unlike caffeine, a specific inhibitor of ATM (KU-55933) was not effective in overcoming the G2 arrest caused by Etoposide (S10D Fig). A specific inhibitor of ATR, VE-821 (S10D Fig), and inhibitors of Chk1/2 (ADZ-7762) or Chk1 (MK-8776), did restore the number of M phase cells when low concentrations of Etoposide were used (S10D and S10E Fig).

**Fig 7:**
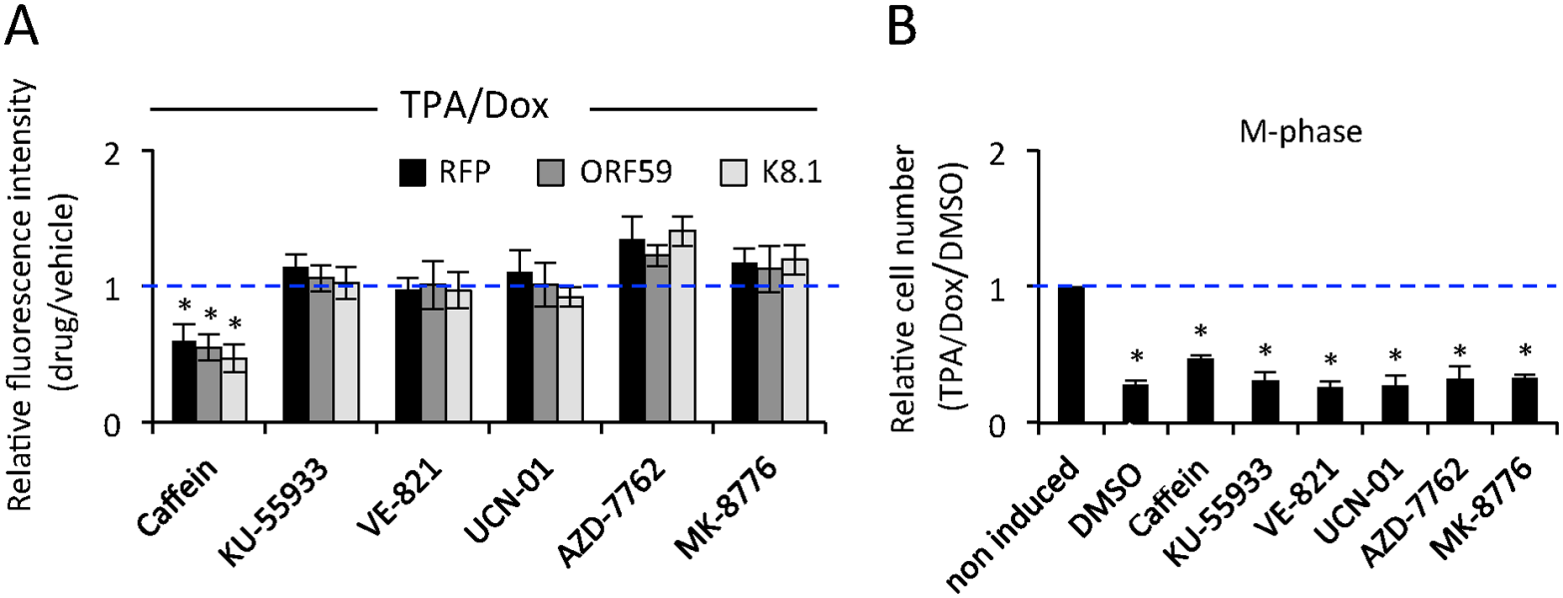
Inhibitors of DNA-damage response have no effect on reactivated cells. (A) Quantification of the expression levels of early (RFP), intermediate (ORF59) and late (K8.1) lytic genes by image analysis of iSLK.219 cells treated with indicated inhibitors of DNA damage response 1 h before reactivation by TPA/Dox, and fixed at 24 h after induction. For each gene, the values were normalized to the fluorescence intensity obtained from reactivated cells treated with vehicle (DMSO, set to one, dashed blue line). The data represent the mean and SD of three independent experiments. (B) Quantifications of the number of cells in M-phase by image analysis of pH3 S10 in iSLK.219 cells treated as in (A). Values represent the mean and SD of three independent experiments and are normalized to the respective non-induced control (set to one, dashed blue line).

Having established the appropriate experimental conditions and the most effective concentrations for each of the inhibitors, we tested their effect in reactivated iSLK.219 cells. Drugs were added 1 h prior reactivation, and maintained in the culture medium throughout the experiment. Cells were fixed 48h after reactivation (TPA/Dox). The extent of viral lytic gene expression and of cell cycle progression was monitored by image analysis of indicated viral lytic genes and pH3- S10, respectively. As shown in Fig 7A, only caffeine partially inhibited the viral lytic gene expression. Consistently, none of the drugs was able to overcome the G2 arrest and restore progression to M-phase (Fig 7B). Our results are consistent with other reports in which the ability of these compounds to sensitize cancer cells to radiation-induced cell death failed if the cells had active p53 and p21 [57].

## Discussion

Through silencing the E3 ubiquitin ligase MDM2 in a siRNA screen we here identify the p53/p21 axis as an important positive regulator of viral reactivation, and demonstrate that cellular stress, an inducer of herpesvirus reactivation, favors KSHV lytic replication. Our data further show that lytic replication leads to severe and sustained DNA damage response and lead to a prominent G2 arrest, indicative of a virus-activated cellular checkpoint. Depletions of p21 in iSLK.219 cells reactivated by TPA/Dox treatment significantly decreased the kinetics of early (RFP, MTA), intermediate (ORF59) and late (K8.1) lytic gene expression and alleviated the G2 cell cycle arrest. Similar results were obtained in biologically relevant PEL cells, BC-3 and BCBL-1. Interestingly, despite the significant reduction in viral lytic gene expression, we observed only a modest decrease in the amounts of infectious virions produced from BCBL1_RTA_ cells after p21 depletion. The modest decrease, however, could be misleading, as compared to p21-depleted cells, the sh-Ctrl expressing cells lysed significantly faster, which could affect the infectivity of the released viruses.

Other DNA viruses, including human papilloma virus, adeno and parvoviruses, have been shown to induce a G2/M arrest during viral DNA amplification [48]. Why would KSHV then benefit from halting the cell cycle at G2? One possibility is that the virus needs to access cellular factors expressed at the G2 phase which could include factors for recombination, repair etc. [58]. Interestingly, we found that reactivated cells were able to bypass the G1/S arrest induced by treatments with Nutlin prior to reactivation. As the G1/S checkpoint is inactivated, the infected cells progress to the S-phase and eventually arrest in G2. While the detailed mechanism of this inactivation is under investigation, we and other groups have demonstrated that the restoration of p53 activity by Nutlin leads to efficient cell death in latent PEL cells and KSHV-infected endothelial cells [44,56,59,60], but fails to kill reactivated cells [61]. The described inactivation of the G1/S checkpoint in cells undergoing lytic replication now explains why the same treatment was not effective in eliminating the reactivated cells [61].

Another advantage of the G2 arrest could be to avoid factors active in M phase that could impair virus amplification. Similar to cellular DNA, the viral DNA genome in infected cells is complexed with nucleosomes [22]. The viral genome may therefore also undergo some degree of modifications (e.g. condensation) when the cell enters mitosis, which could disturb viral lytic gene expression and DNA replication once the lytic cycle is initiated. While more experiments are required to address this possibility, a recent report showed that KSHV genome can be co-immunoprecipitated using antibodies against pH3-S10 [17], a modification present during chromatin condensation at the onset of mitosis [55,62–65]. Interestingly, depleting the enzymes that are responsible for H3 phosphorylation at S10 led to robust spontaneous reactivation and decreased levels of pH3-S10 on the viral genome [17]. Both p21 and the DNA-damage-activated Chk1, which we found active during reactivation, can inhibit the G2/M transition [66–68]. p21 has been shown to efficiently inhibit CDK1 in response to DNA damage, which was sufficient to cause a permanent G2 arrest [69]. The early induction of p21 described here could therefore inhibit Cdk1, support the G2 arrest and increase the efficiency of virus reactivation. Intriguingly and supporting this hypothesis, Cdk1 inhibition by small molecule inhibitors has been shown induce spontaneous KSHV reactivation in PEL cells [70].

Although the activation of p21 seems to be an early event during KSHV reactivation, the activation of a DNA damage response (e.g. pATM, pChk1) observed at later stages of the lytic cycle could reinforce the inhibition of the cell cycle progression [66]. We therefore attempted to inhibit the DDR by small molecule inhibitors, hoping to restore the cell cycle progression and activate cell death responses during M phase, a process known as ‘mitotic catastrophe’ [71,72]. This strategy has been successfully used to sensitize cancer cells to radiation-induced DNA damage [73]. However, the DDR inhibitors have not been effective in cells with functional p53/p21 axis, because cell death is prevented by the p53-dependent cell cycle arrest [74,75]. Our attempt of using inhibitors of DDR to induce cell death in reactivated cells was also unsuccessful. Similar to other cancer cells, this resistance could be due to p21-induced cell cycle arrest.

The tumor suppressor p53 is often mutated and inactivated in human cancers. However, this is not the case in KSHV-associated malignancies, where p53 mutations are rarely found [39,59,76]. Instead, the virus has co-evolved with wild type p53 and utilizes a large repertoire of mechanisms to bypass its growth restrictive functions during the latency [38–42,44,45,59]. Our work now provides a mechanistic explanation as to why the virus has evolved to retain p53 and p21 activities to support the lytic phase.

Our ChIPSeq cell analyses indicated that p53 does not bind KSHV genome during the reactivation. Work on murine gammaherpesvirus 68 (MHV68) and Epstein Barr virus (EBV), two other gammaherpesviruses, has demonstrated that p53 contributes to the expression of the lytic genes, by stimulating transcription of RTA or RTA and an immediate-early viral Zta gene, respectively [77–79]. Our results now demonstrate a different role for p53 in KSHV reactivation, where this transcription factor is required to enhance viral replication through modulation of the host cell stress responses induced upon viral lytic replication.

## Materials and Methods

Cells, plasmids, antibodies, primers, and standard methods are described in SI Materials and Methods.

### shRNA and siRNA treatments

Lentiviral expression plasmids pLKO.1-sh-Scr, sh5-MDM2, sh1-p53, sh2-p53, sh1-p21 and sh2-p21 were obtained from Open Biosystems and Biomedicum Functional Genomics Unit (Helsinki, Finland). Lentivirus stocks were produced as described [56]. To establish BC-3 cell lines stably expressing sh-p53, cells were selected with medium containing 3.5 μg/ml puromycin (Sigma) and to establish TREx BCBL1-Rta (here referred as BCBL1_RTA_) and BC-3 cell lines stably expressing sh-p21, cells were selected with medium containing 2 μg/ml puromycin (Sigma).

The nonspecific siRNAs control (ON-TARGETplus, D-001810-10) and siRNA against p21 (ON-TARGETplus, L-003471-00) were from Dharmacon (SMARTpool siRNA). Cells were reverse-transfected (2000 cells per well) for 48 h in 96-well view plates (Perkinelmer) using RNAiMax (Invitrogen), and reactivated by indicated treatments for 24 h. For more details see supplemental information.

### Cell spot microarray siRNA screen

The Primary screen was performed with the cell spot microarray technique [33]. 48 h after reverse transfection, rKSHV-SLK cells were reactivated for 30h, fixed and imaged with an Olympus Scan-R high-content microscope. For more details see S1 Methods.

### Immunofluorescence and western blot

For immunofluorescence in 96 well imaging plates (Perkin Elmer) cells were fixed in PBS containing 4% paraformaaldehyde, (20 min, room temperature), permeabilized for 10 min in PBS containing 0.1% Triton-X 100 (Sigma). All antibody incubations were performed in PBS containing 2% BSA for 45 min. Alexafluor-conjugated secondary antibodies were from Invitrogen.

For western blot analysis cells were lysed in RIPA buffer containing protease (Thermo scientific; Cat # 88666) and phosphatase inhibitors (Thermo scientific; Cat # 88667) and whole cell extracts were then clarified by centrifugation, mixed with Laemmli buffer and boiled for 5 min. Protein concentration was determined by Bio-Rad protein assay (Bio-Rad) and 20–40 μg of protein were loaded in each lane of a Criterion TGX midi gel 4–15% (Bio-Rad) and transferred to nitrocellulose membranes (Protran nitrocellulose membrane 0,45 um, Perkin Elmer). Membranes were immunostained in TBST buffer containing 5% fat-free milk, and HRP-conjugated secondary antibodies detected with the Enhanced Chemiluminescence kit (Western Bright Sirius, Advansta) on Fuji films. Densitometry analysis was performed using the ImageJ software (National Institutes of Health, USA).

### ChIP and ChIP-seq experiment

ChIP and sequencing were carried out as described [80] using a monoclonal antibody against p53 (Clone DO-1, GeneSpin) and an Illumina HiSeq 2000 (single 36 bp reads) system. The analysis of the sequencing data was performed as described [81]. For details see S1 Methods.

### Drug treatments

Caffeine (Sigma) was dissolved in water. All the other drugs were dissolved in DMSO and stored at -20 C. Nutlin-3, Etoposide, KU-55933 and UCN-01 (Sigma); MK-8776 (also called SCH900776, Chemie Tech); VE-821 and AZD-7762 (Selleckchem). All experiments were performed in 96-well imaging plates (PerkinElmer). For details see S1 Methods.

### High content imaging and image analysis

All experiments were performed in 96-well imaging plates (Perkin Elmer) and images acquired using the automated fluorescence microscope Cellinsight (Thermo Scientific). Image analysis was performed with the Cell Profiler 2 software [82]. For the analysis of the pH3 S10 staining we modified a premade imaging pipeline ("Percent positive") available in the Cell Profiler web site (www.cellprofiler.org). After detection of nuclei, the G1/S, G2 and M-phase cells were classified by thresholding the mean fluorescence intensity of pH3 S10 signal in each nucleus.

### FACS analysis of cell cycle progression

For FACS analysis cells were fixed/permeabilixed in 70% Ethanol at -20°C for 3 h, washed once with PBS and incubated for 45 min in PBS containing 30 μg/ml RNase and 10 μg/ml propidium iodide. Cells were washed two times with PBS before FACS analysis. For each condition, 10.000 cells were analyzed.

### Accession numbers

MDM2 (Gene ID: 4193); TP53 (Gene ID: 7157); p21(CDKN1A; Gene ID: 1026); P53R2 (Gene ID: 50484); PAG608 (Gene ID: 64393); Cyclin B1(CCNB1; Gene ID: 891); H2AX (Gene ID: 3014); ATM (Gene ID: 472); ATR (Gene ID: 545); CHK1 (Gene ID: 1111); CHK2 (Gene ID: 11200) KSHV genes: ORF73/LANA (Gene ID: 4961527); ORF50/RTA (Gene ID: 4961526); ORF57/MTA (Gene ID: 4961525); ORF74/vGPCR/K14 (Gene ID: 4961465); ORF25 (Gene ID: 4961452); ORF29 (Gene ID: 4961443); K8.1 (Gene ID: 4961469)

## Supporting Information

S1 Methods Description of the following reagents and methods used in the article: Cells, Plasmids and lentivirus vectors, Cell spot microarray siRNA screen, WB, ChIP-seq, Quantitative Real time PCR, and Virus release assay from BCBL-1_RTA_ cells. (DOCX)

S1 Fig Targeted RNAi screen to identify epigenetic cell factors that regulate KSHV reactivation. (A) Experimental and analysis workflow of the siRNA screen using rKSHV-SLK cells and the cell-spot microarray (CSMA) technology (experimental details in materials and methods). (B) Distribution of the Z-scores for all 662 genes included in the siRNA screen. The values obtained using the two siRNAs against MDM2 are indicated by the yellow arrowhead. siRNAs that resulted in changes of RFP intensity larger then 2xSTDEV (-2> Z >2) from the median of all values of the screen were considered as hits (above and below the yellow and red dashed lines, respectively).
(TIF)

S2 Fig Characterization of the efficiency of chemically-induced lytic reactivation in SLK.219 cells. (A) Induction of RTA (green) in iSLK.219 cells treated with control DMSO or doxycycline (Dox, 0.4 ng/ml) for 24 hours. RTA was detected after immunofluorescence staining using anti-RTA antibodies. The RFP (red) expression indicates virus lytic reactivation. Nuclei (grey) were counterstained with Hoechst. (B) Induction of reactivation (RFP, red) in iSLK.219 cells treated with DMSO control or TPA (20 ng/ml), Dox (0.4 ng/ml), NaB (1.32 mM) or a combination of Dox and TPA (TPA/Dox) or Dox and NaB (NaB/Dox) for 24 hours. The right-most panels indicate the RFP intensity (displayed in 'Fire' color with Image-J). (C) Automated image analysis after high-content imaging was used to quantify the median RFP fluorescence intensity and the fraction of RFP positive cells in iSLK.219 cells treated as indicated. For each condition, 16 images and more the 1500 cells were analyzed. Error bars represent the SD of three independent experiments.
(TIF)

S3 Fig KSHV reactivation induces a bona fide p53 response in PEL cells. (A) Graphical representation of the average sequencing signal obtained after ChIP-seq of BC-3 cells treated with vehicle (DMSO) or TPA for 24 h. As a negative control, a nonspecific IgG antibody was used. The coverage of ChIP-seq reads extended to the fragment length (with duplicate reads removed) of each sample was calculated for each of the top 99 peaks called from the 24 h sample. The coverage curves were averaged over all peak regions for each sample separately. Finally, the coverage values were normalized to million reads mapped. The graph also includes the background signal from the nonspecific IgG controls (yellow and light blue). (B) Heat-map of the strongest peaks and associated genes identified after ChIP-seq analysis of BC-3 cells treated with DMSO, TPA (24 h) or Nutlin (8 h). The scale has been normalized to reads per peak per million mapped reads. The red arrowheads indicate genes that were associated with similar (or higher) number of reads in cells treated with TPA compared with those obtained from cells treated with Nutlin. (C) Schematic representation of gene-pathways enriched in response to p53 activation after DAVID enrichment analysis. The asterisks indicate the genes identified from the ChIP-seq analysis of TPA (red) or Nutlin (blue) treated cells. Indicated in the scheme are representative genes pooled out from the top 300, statistically significant (p<0.05), peaks in each of the two treatments. (D) The Venn diagrams display the number of peak regions called from the 24 hour TPA sample (N = 99), overlapping with the top 1054 most significant peak-regions (lowest p-values) obtained from Nutlin treated cells. (E-F) iSLK.219 cells (E) or non-infected SLK cells (F) were treated with vehicle (DMSO) or indicated inducers for 4 h (iSLK.219) and 12 h (SLK) and processed for WB using antibodies against p21, GAPDH and also MTA for iSLK.219.
(TIF)

S4 Fig Depletion of p53 impairs the expression of lytic genes in BC-3 cells. (A) Fluorescence images showing the stabilization of p53 after Nutlin treatment in iSLK.219 treated for 24h and processed for immunofluorescence imaging using antibodies against p53 (green) and hoechst to visualize nuclei (grey). (B) Inhibition of cell growth in iSLK.219 cells treated with Nutlin for 24h. Hoechst-stained nuclei were counted by automated image analysis after high-content fluorescence imaging. Values obtained from Nutlin treated cells were normalized to the cell number obtained in the respective DMSO treated sample. Shown are the average values obtained from three independent experiments. The error bars represent SD. More than 1500 cells were counted in each repetition. (C) mRNA levels of indicated viral lytic genes in BC-3 cells stably expressing sh-Ctrl or sh1-p53 and treated with TPA for indicated times. For each time point, the results are normalized to the values obtained from cells stably expressing the non-specific sh-Ctrl. Error bars represent the SEM. * P<0.05. (D) mRNA levels of indicated viral lytic genes in BC-3 cells stably expressing sh-Ctrl, sh1-p53 or sh2-p53, and treated with NaB for 24 h. For each treatment, the results are normalized to the values obtained from cells stably expressing the non-specific sh-Ctrl and treated with the same inducer. Error bars represent the SEM. * P<0.05. (E) BC-3 cells stably expressing sh-Ctrl or sh1-p53 were treated with TPA for indicated times (hpi). The levels of viral lytic gene expression (MTA), p53 and p21 were analyzed by WB using the respective antibodies. Tubulin was used as a loading control. Note that at 4 hpi, the levels of p21 are much reduced in cells expressing sh1-p53 compared to sh-Ctrl, in the respective time point (red dashed box).
(TIF)

S5 Fig Depletion of p21 in BC-3 and BCBL1_RTA_ cells has no effect on cell growth. (A-B) Growth curve of BC-3 (A) and BCBL1_RTA_ (B) cells stably expressing indicated shRNA. Cells were counted at indicated time points with a BioRad TC20 automated counter. (C-D) Efficiency of p21 depletion monitored by qRT-PCR in BC3 (C) and BCBL1_RTA_ (D) cells stably expressing sh-Ctrl, sh1-p21 or sh2-p21. Values represent the mean and SDM of three independent experiments and are normalized to sh-Ctrl. (E) Fluorescent images after high-content imaging of U2OS cells infected for 48h with viruses released from the BCBL-1_RTA_ sh-Ctrl cells treated with DMSO or reactivated by Dox, and processed for IF using antibodies against ORF 73 (LANA). The lower panels represent higher magnifications of the respective white-boxed areas. Arrowheads in each image indicate LANA-positive (green arrowhead) of LANA-negative (white arrowhead) cells. (F) Quantification of the fraction of infected (LANA-positive) cells in U2OS cells infected as in (E) with viruses collected from the indicated cell lines. Each value represents the mean and STDEV of three independent experiments. * p<0.05.
(TIF)

S6 Fig Image-based analysis of the cell cycle progression in iSLK.219. (A) Automated image analysis was performed with the Cell Profiler software to identify an area corresponding to the Hoechst stained nuclei of each cell (purple lines in the left-most image) and, within this area, quantify the intensity of fluorescence of pH3 S10 detected after immunofluorescence staining and imaging (indicated in red in the middle image). Based on the fluorescence intensity of the pH3 S10, cells were classified into G1/S (no signal, blue nuclei), G2 (low intensity, light-green nuclei) and M (bright fluorescence, red nuclei). (B) Fluorescence images of non-induced DMSO-treated iSLK.219 cells after immunofluorescence analysis using antibodies for pH3 S10 (green) and Cyclin B1 (red). The different stages of the cell cycle are indicated by italics letters as in Fig 4A. (C) Digital images after analysis of cell cycle progression with Cell profiler as in A. iSLK.219 cells were treated with either DMSO (0.1%), Nutlin-3A (10 mM, 48h), etoposide (6.25 mM, 24 h) or TPA/Dox for 24 h before pH3 S10 immunostaining, high-content imaging and image analysis. (D) Quantification of cells in M- or G2-phase by image analysis in iSLK.219 cells treated as in C. The values represent the mean and SD of three independent experiments. In each experiment more than 1500 cells were analyzed. For each treatment, values are normalized to the respective DMSO controls (set to one, dashed red line).
(TIF)

S7 Fig Correlation between pH3 S10 and Cycling B1 in reactivated G2 arrested cells. (A) Cross-talk between fluorescent channels. Fluorescence images of iSLK.219 cells treated with TPA/Dox for 20 h and processed for immunofluorescence analysis in the absence of primary antibody and using only the secondary Alexa-647 conjugated antibodies. No fluorescence signal is detected in the far-red channel (no Ab) with the same imaging conditions as in the experiments where the antibody against pH3 S10 was included (compare with Fig 4B). (B-C) Fluorescence images of non-induced (DMSO, C) or induced (TPA/Dox, D) iSLK.219 cells after immunofluorescence analysis using antibodies for Cyclin B1 (green). The RFP expression (red) indicates cells that undergo lytic reactivation. The right-most images are higher magnifications of the white-boxed areas. Arrowheads indicate reactivated cells that are positive for RFP and Cyclin B1.
(TIF)

S8 Fig FACS analysis of cell cycle progression in reactivated iSLK.219 cells. (A) Fluorescence intensity of GFP (left panel) and RFP (right panel) in iSLK.219 cells treated with DMSO (non induced, red line) or TPA/Dox (reactivated, blue line). (B) Cell cycle analysis of iSLK.210 cells treated with indicated drugs for 24 h and stained with PI. (C) Cell cycle analysis of iSLK.210 cells treated with TPA/Dox for 24 h and stained with PI. (D) Comparison of fluorescence signal between TPA/Dox treated iSLK.219 cells stained (red line) or not (blue line) with PI. (E-G) Quantification of cell cycle distribution in iSLK.219 cells treated with the indicated compounds for 24 h and stained with PI. Values represent the mean and SD of three independent experiments. The total number of counted cells in each condition is expressed as 100%.
(TIF)

S9 Fig Non-infected SLK cells cannot by-pass the Nutlin-induced G1/S arrest. (A) Fluorescent images of SLK cells treated with indicated drugs for 48 h and processed for IF as in Fig 6, using antibodies to detect pH3 S10 (green) and Hoechst to detect nuclei (grey). The lower panels represent higher magnifications of the respective white-boxed areas. Images are the representative of three independent experiments.
(TIF)

S10 Fig Inhibitors of DNA damage response induce cell death in iSLK.219 cells treated with etoposide. (A) Schematic representation of the DDR and inhibitors (purple) used in this study. (B) Fluorescence images of iSLK.219 cells treated with H_2_O (control) or caffeine (5 mM) 1 h prior the addition of etoposide (12.5 μM). Cells were fixed 48 h later and processed for immunofluorescence analysis using antibodies against pH3 S10 (green) and γH2AX (red). Nuclei are stained with Hoechst (grey). The lower panels are higher magnifications of the respective white-boxed areas (numbered 1,2 and 3). (C) The ability of Caffeine to inhibit the DDR and restore the cell cycle progression from G2 to M-phase was analyzed by image analysis as described in S5 Fig. iSLK.219 cells were treated with H_2_O (control) or indicated concentrations of caffeine for 1 h before the incubation with etoposide for 48 hours. For each treatment, values represent the mean and SD of three independent experiments, and are normalized to the number of M-phases detected in H_2_O treated cells (set as 1, dashed red line). (D-E) The abilities of different inhibitors of DDR to prevent G2-arrest and restore M-phases were tested as in C. For each treatment, values represent the mean and SD of three independent experiments, and are normalized to the number of M-phases detected in DMSO treated cells (set as 1, dashed red line).
(TIF)
