# Supplementary material for: Oncogenic Herpesvirus Utilizes Stress-Induced Cell Cycle Checkpoints for Efficient Lytic Replication
Source: PLoS Pathog. 2016 Feb 18;12(2):e1005424. doi: 10.1371/journal.ppat.1005424 (PMC4758658; doi:10.1371/journal.ppat.1005424)
Supplement: S1 Methods — (DOCX) [file ppat.1005424.s001.docx]

# Supplementary Methods

**Cells**

rKSHV.219 infected SLK cell line (rKSHV-SLK) was established and maintained as described in [44]. **RTA-inducible SLK cells (iSLK.219) harboring recombinant KSHV (rKSHV.219)** were kindly provided by J. Myoung and D. Ganem (Novartis, CA, USA) and maintained as described [35]. BC-3 cells were obtained from the National Institutes of Health AIDS Research and Reference Reagent Program (catalog # 3233 from McGrath and Ganem) and cultured as described previously [44]. TREx BCBL1-Rta cells were a kind gift from J. Jung (Keck School of Medicine, USC, CA, USA).

To induce KSHV lytic replication, rKSHV-SLK cells were treated as described [44], iSLK.219 cells were treated 0.2 μg/ml doxycycline and 1.35 mM sodium butyrate (NaB; Sigma, St Louis, MO) or doxycyclin and 20 ng/ml 12-O-tetradecanoylphorbol-13-acetate (TPA; Sigma); BC-3 cells were treated with 1.35 mM NaB or 20 ng/ml TPA and TREx BCBL1-Rta cells with 0.2 µg/ml doxycycline.

**Plasmids and lentivirus vectors**

For production of shRNA-expressing lentiviruses the following LKO.1 plasmids were used(shRNA sequence):

pLKO.1-sh-Scr

(CCTAAGGTTAAGTCGCCCTCGCTCAGCGAGGGCGACTTAACCTTAGG)

sh5-MDM2

(CCGGCTCAGCCATCAACTTCTAGTACTCGAG-TACTAGAAGTTGATGGCTGAGTTTTT)

sh3-MDM2 (CCGGCGATTATATGATGAGAAGCAACTCGAGTTGCTTCTCATCATATAATCGTTTTT)

sh1-p53 (CCGGCGGCGCACAGAGGAAGAGAATCTCGAGATTCTCTTCCTCTFTGCGCCGTTTTT)

sh2-p53 (CCGGGAGGGATGTTTGGGAGATGTACTCGAGTACATCTCCCAAACATCCCTCTTTTT)

sh1-p21

(GCTGATCTTCTCCAAGAGGAA)

sh2-p21

(AGAGGTTCCTAAGAGTGCTGG)

## Lentivirus stocks were produced as described [24]. Lentiviral supernatants were concentrated by ultracentrifugation at 22.000 rpm (SW-32-Ti rotor (Beckman Coulter), +4°C. To silence expression of *MDM2*, *TP53 or p21*, BC-3 or TREx BCBL1-Rta (for depleting *p21*) cells were seeded at density 5x10^5^ cells/ml and transduced in the presence of 8 µg/ml polybrene (Sigma) in a 50 ml culture flask using for each shRNA of the corresponding lentivirus stock. To establish cell lines stably expressing sh-p53, cells were selected with media containing 3.5 μg/ml puromycin and for sh-p21 with media containing 2 μg/ml puromycin (Sigma).

**Cell spot microarray siRNA screen**

The custom-made siRNA library (Qiagen), targeting genes with Gene Ontology (GO) annotations related to epigenetics, chromatin remodelling/maintenance, and co-regulatory functions (described in [32] was complexed with lipid transfection agents and Matrigel (BD Biosciences) and spotted/arrayed onto an untreated hydrophobic polystyrene surface. rKSHV-SLK cells were seeded onto the array as a 30.000 cells /ml suspension, and allowed to adhere for 15 min. Unattached cells were then washed off. After 48 hours the cells were treated with recombinant baculovirus (BacK50) expressing the KSHV lytic activator ORF 50 (RTA; a gift from J. Vieira; University of Washington) for 2 h at room temperature and 6 hours in +37°C. The use of baculoviruses to express RTA was necessary since SLK cells do not reactivate with only TPA or NaB treatments. The more recent Doxycyclin-inducible RTA system, the iSLK.219 cells [35], had not been yet released when the screen was performed.

As a positive control for maximal reactivation, one array was infected with RTA-expressing baculoviruses and 1.35 mM sodium butyrate (Sigma), which increase the efficiency of reactivation.

Following baculovirus infection, cells were incubated for additional 30 hours, fixed with 4% paraformaldehyde (PFA; Electron Microscopy Sciences) and processed for the analysis of GFP (marker of latent infection) or RFP (marker of lytic reactivation) expression using a LS400 laser scanner (Tecan) to obtain a single low resolution image of the whole array. From this image, the fluorescence of each spot was quantified with the ArrayPro Analyzer v. 4.5 (Media Cybernetics Inc) to obtain the mean intensity fluorescence value of the whole screen. Total raw mean intensities of signals for all cells per spot were normalized using pin normalization to obtain the net intensities. The net intensity value of each spot was then compared to that of the mean of the screen, used as a control, and a Z-score was calculated for each siRNA treatment. From this analysis, siRNAs with Z-scores >2 s.d. were considered significant and cells re-imaged separately using an Olympus Scan-R high content microscope (Olympus Europe, Munich, Germany) for phenotype validation.

For ‘hit’ validation, iSLK.219 cells were plated on 96-well view plates (Perkin Elmer) at a density of 3000 cells/well one day before siRNA transfection. Cells were the transfected with nonspecific control or siRNA oligos against *MDM2 (*4 siRNA oligos per gene, Qiagen) using the DharmaFECT1 transfection reagent (Dharmacon) according to the manufacturer’s instructions. To obtain the maximal sensitivity of the assay, 24 hours after transfection the cells were treated with a suboptimal dose (0.05 μg/ml) of doxycycline, which results in low levels reactivation. After 52 hours cells were fixed with 4% PFA and the induction of RFP expression was monitored with an automated high-content fluorescence microscope (CellInsight, Thermo Scientific).

**WB**

For western blot analysis cell were lysed in RIPA buffer containing protease and phosphatase inhibitors (Roche, cat numb.) ELB lysis buffer (150 mM NaCl_2_; 50 mM HEPES, pH 7.4; 0.1% NP-40; 5mM EDTA; 2 mM DTT; Pierce Protease inhibitor mini tablet, EDTA-free (Thermo scientific)) and whole cell extracts were then clarified by centrifugation at 17.000xg for 20 minutes at 4°C. Total protein concentration was determined by Bio-Rad protein assay (Bio-Rad) and 20-40 μg of whole cell extracts were separated by Criterion TGX midi gel 4-15% (Bio-Rad) and transferred to nitrocellulose membrane (Protran nitrocellulose membrane 0,45 um, PerkinElmer). Immunoblotting was carried out by using an anti-Mdm2 antibody mixture (IF2 (Calbiochem,), SMP14 and 2A10 (Abcam)), anti-RTA (a kind gift from B. Chandran, Rosalind Franklin University and Yan Yuan, University of Pennsylvania) anti-ORF57 (sc-135746, Santa Cruz Biotechnology), anti-p53 (FL-393, Santa Cruz Biotechnology), anti-p21 (C-19, sc-397 Santa Cruz Biotechnology), and anti-tubulin (GTU-88, Sigma). The membranes were probed for protein detection by enhanced chemiluminescence (Western Bright Sirius, Advansta). Quantitative analysis was performed using the ImageJ software(National Institutes of Health, USA).

**ChIP-seq**

BC-3 cells were cross-linked with 1% formaldehyde in PBS at room temperature for 10min. After a wash in PBS containing 125 mM glycine, cells were collected by centrifugation and sonicated in lysis buffer containing 0,1% SDS, 0,1% sodium deoxycholate, 1 mM EDTA, 10 mM TrisHCl pH 8.0, 140 mM NaCl, 1% Triton X-100 and protease inhibitors to generate chromatin fragments of 100–300 bp in length. Following a brief centrifugation (16.000xg, 20 min, 4 °C), the fragmented chromatin was immunoprecipitated with a monoclonal antibody against p53 (Clone DO-1, GeneSpin) or control IgG (normal mouse IgG: sc-2025, Santa-Cruz Biotechnology). The precipitates were incubated at 65 °C over night to reverse the formaldehyde crosslinking and samples incubated with proteinase K RNase-A before RNA phenol-extraction and ethanol-precipitation. The precipitated DNA was first repaired using Klenow and T4 DNA polymerases and T4 polynucleotide kinase (MBI Fermentas, Latvia), and then ligated to adapters according to manufacturer's instructions (Illumina). PCR-amplified fragments of approximately 180-300bp were sequenced using an Illumina HiSeq 2000 (single 36 bp reads) system. The analysis of the sequencing data was performed as described [86].

**Quantitative Real time PCR**

Total RNA was prepared by using the RNAeasy kit (Qiagen, Valencia, CA) according to manufacturer instructions. RT-PCR was performed with the TaqMan Reverse Transcription Reagents kit (Roche Diagnostics, Indianapolis, IN). Real-time PCR conditions and primers are described in supplemental materials and methods.

Primers for ORF50, ORF57, K8.1 and human beta-actin used in this study have been described [24].

Primers for the other analyzed genes:

LANA (ORF73) sense: 5´- ACTGAACACACGGACAACGG-3´;

antisense: 5´- CAGGTTCTCCCATCGACGA-3´

 vGPCR (ORF74): sense: 5´- CGCTGCACTGTTAATTGCAT -3´;

antisense: 5´- GTCGCCTTAGCAGAGTGTCC -3´

 ORF25: sense: 5´- GTCCACCCCTTCTTTGATTTTT -3´;

antisense: 5´- TTTCCCGAGTTGACCCAGTAGG -3´

ORF29: sense: 5´- CCCGGAGGACGGTCCA -3´;

antisense: 5´- CCCCGAATGCTCTGTTCTTATT -3´

K8.1: sense: 5´- AAAGCGTCCAGGCCACCACAGA -3´;

antisense: 5´- GGCAGAAAATGGCACACGGTTAC -3´

p21: sense: 5´- GGCAGACCAGCATGACAGATT-3´;

antisense: 5´- GCGGATTAGGGCTTCCTCT-3´

For statistical analysis of qRT-PCR data was analysed as in [24].

**Virus release assay from BCBL-1_RTA_ cells**

Viruses released from the indicated BCBL-1_RTA_ cells (grown in the absence of puromycin for 24h) were collected from supernatants (30 ml) of cells (5x10^5^/ml) reactivated by doxycycline (0.2 μg/ml) for 24 hours. Supernatants were cleared by centrifugation at 300 x g for 5 min, and followed by filtration (0.45 μm filters). Viruses were concentrated by centrifugation at 100.000xg, 120 min, 4 °C, on a SW-28 rotor (Beckmann). Pellets were resuspended in 100 µl RPMI. A serial dilution of this virus preparation was immediately used to spin-infect U2OS cells (5000 cells/well) in 96- well imaging plates (Perkin Elmer) (450 x g for 15 minutes at 23 °C) in RPMI supplemented with 0.2% BSA, 20mM HEPES, pH 7.2 and glutamine and placed in an incubator at 37 °C and 5% CO_2_. Five hours after infection the inoculum was removed and cells grown in the same medium containing 10% FCS for 48h before standard fixation in 4% PFA and IF analysis using antibodies to detect ORF 73 (LANA). After high-content imaging (Cellinsight microscope, Thermo Scientific), the fraction of LANA-positive cells in each well was determined using automated image analysis with CellProfiler. Approximately 1000 cells were counted in each well (16 images per well using a 20x objective).
