# Supplementary figures and images for: Oncogenic Herpesvirus Utilizes Stress-Induced Cell Cycle Checkpoints for Efficient Lytic Replication
Source: PLoS Pathog. 2016 Feb 18;12(2):e1005424. doi: 10.1371/journal.ppat.1005424 (PMC4758658; doi:10.1371/journal.ppat.1005424)

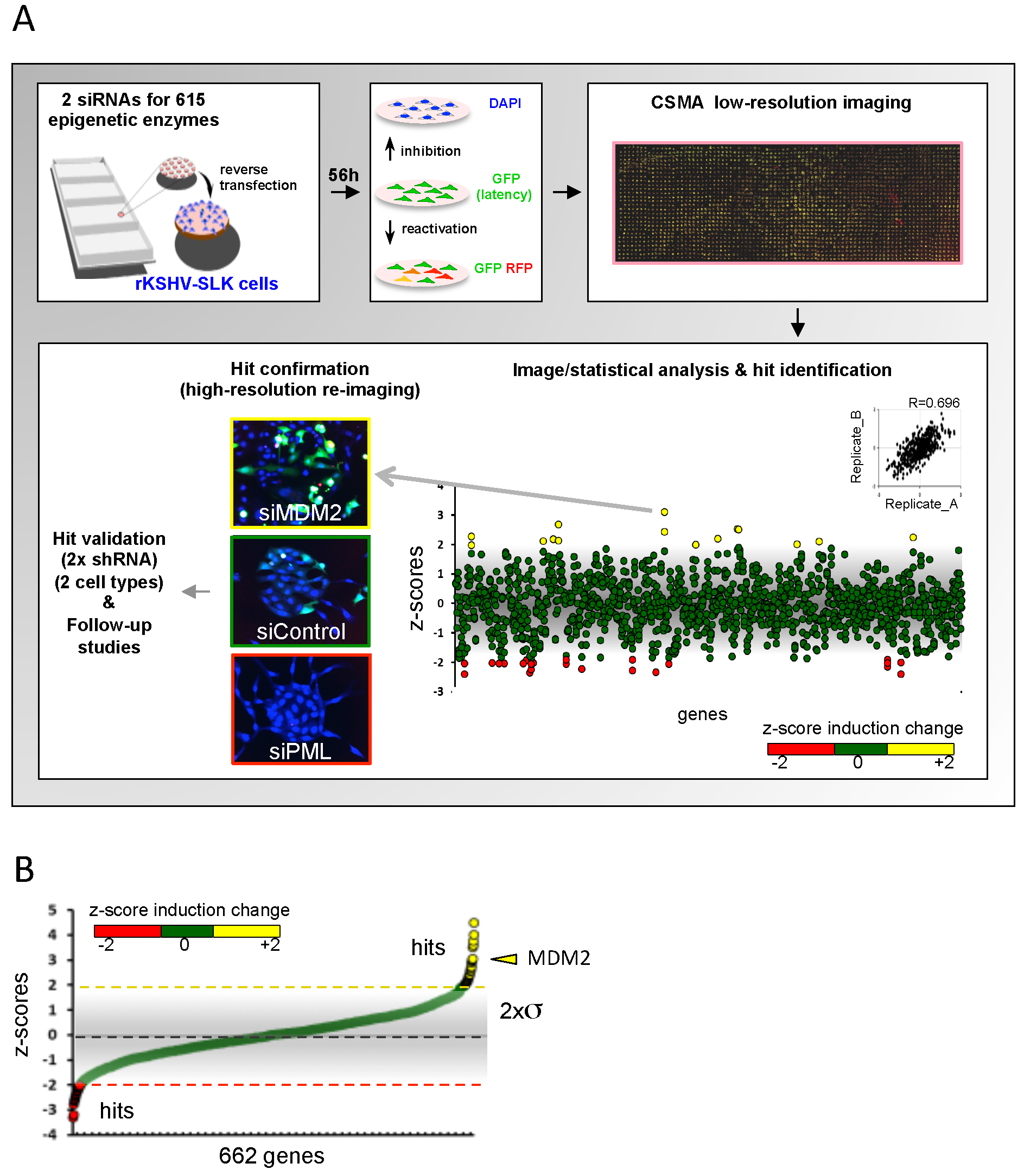

Supplement: S1 Fig — (A) Experimental and analysis workflow of the siRNA screen using rKSHV-SLK cells and the cell-spot microarray (CSMA) technology (experimental details in materials and methods). (B) Distribution of the Z-scores for all 662 genes included in the siRNA screen. The values obtained using the two siRNAs against MDM2 are indicated by the yellow arrowhead. siRNAs that resulted in changes of RFP intensity larger then 2xSTDEV (-2> Z >2) from the median of all values of the screen were considered as hits (above and below the yellow and red dashed lines, respectively). (TIF) [file ppat.1005424.s002.tif]

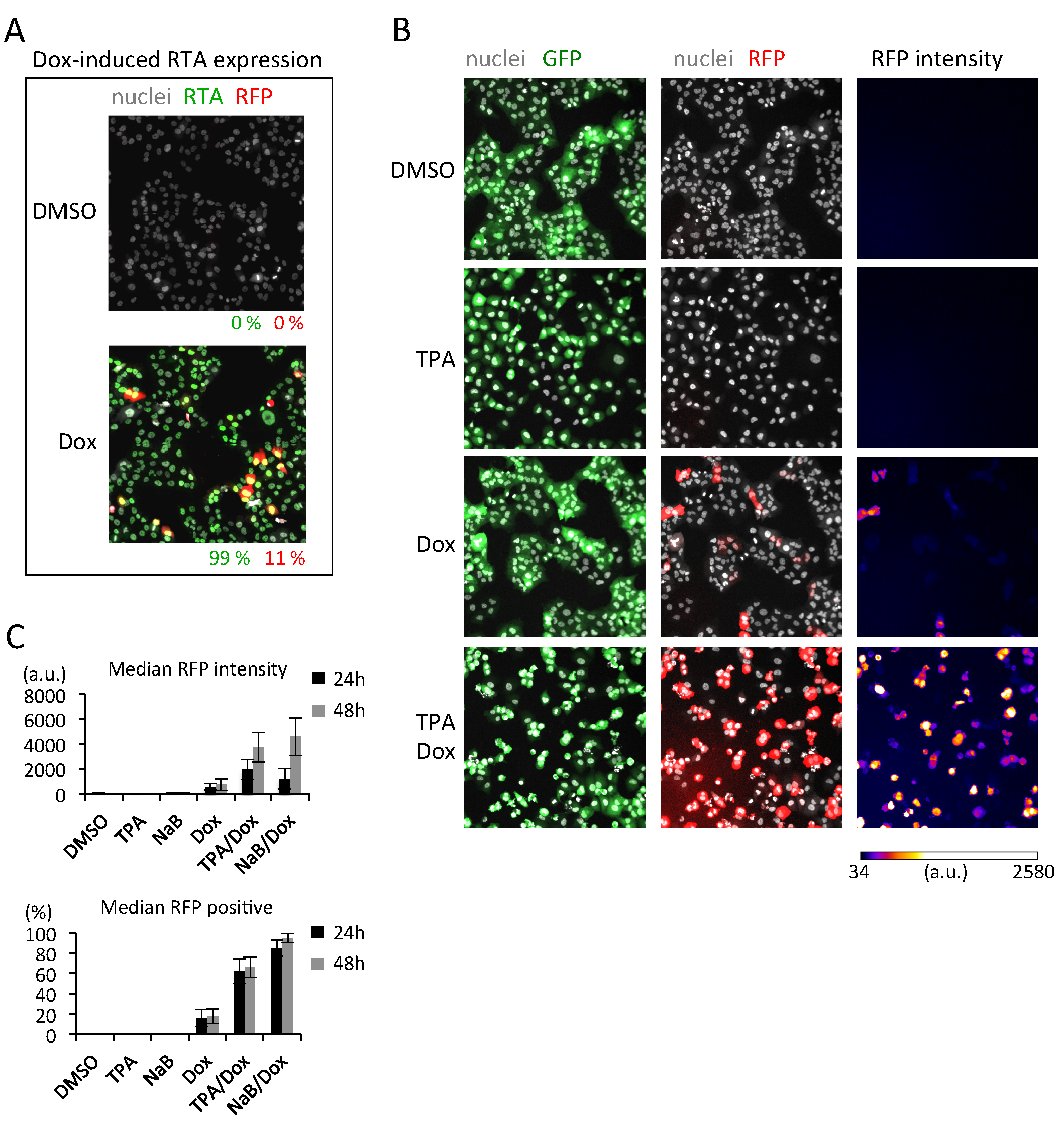

Supplement: S2 Fig — (A) Induction of RTA (green) in iSLK.219 cells treated with control DMSO or doxycycline (Dox, 0.4 ng/ml) for 24 hours. RTA was detected after immunofluorescence staining using anti-RTA antibodies. The RFP (red) expression indicates virus lytic reactivation. Nuclei (grey) were counterstained with Hoechst. (B) Induction of reactivation (RFP, red) in iSLK.219 cells treated with DMSO control or TPA (20 ng/ml), Dox (0.4 ng/ml), NaB (1.32 mM) or a combination of Dox and TPA (TPA/Dox) or Dox and NaB (NaB/Dox) for 24 hours. The right-most panels indicate the RFP intensity (displayed in 'Fire' color with Image-J). (C) Automated image analysis after high-content imaging was used to quantify the median RFP fluorescence intensity and the fraction of RFP positive cells in iSLK.219 cells treated as indicated. For each condition, 16 images and more the 1500 cells were analyzed. Error bars represent the SD of three independent experiments. (TIF) [file ppat.1005424.s003.tif]

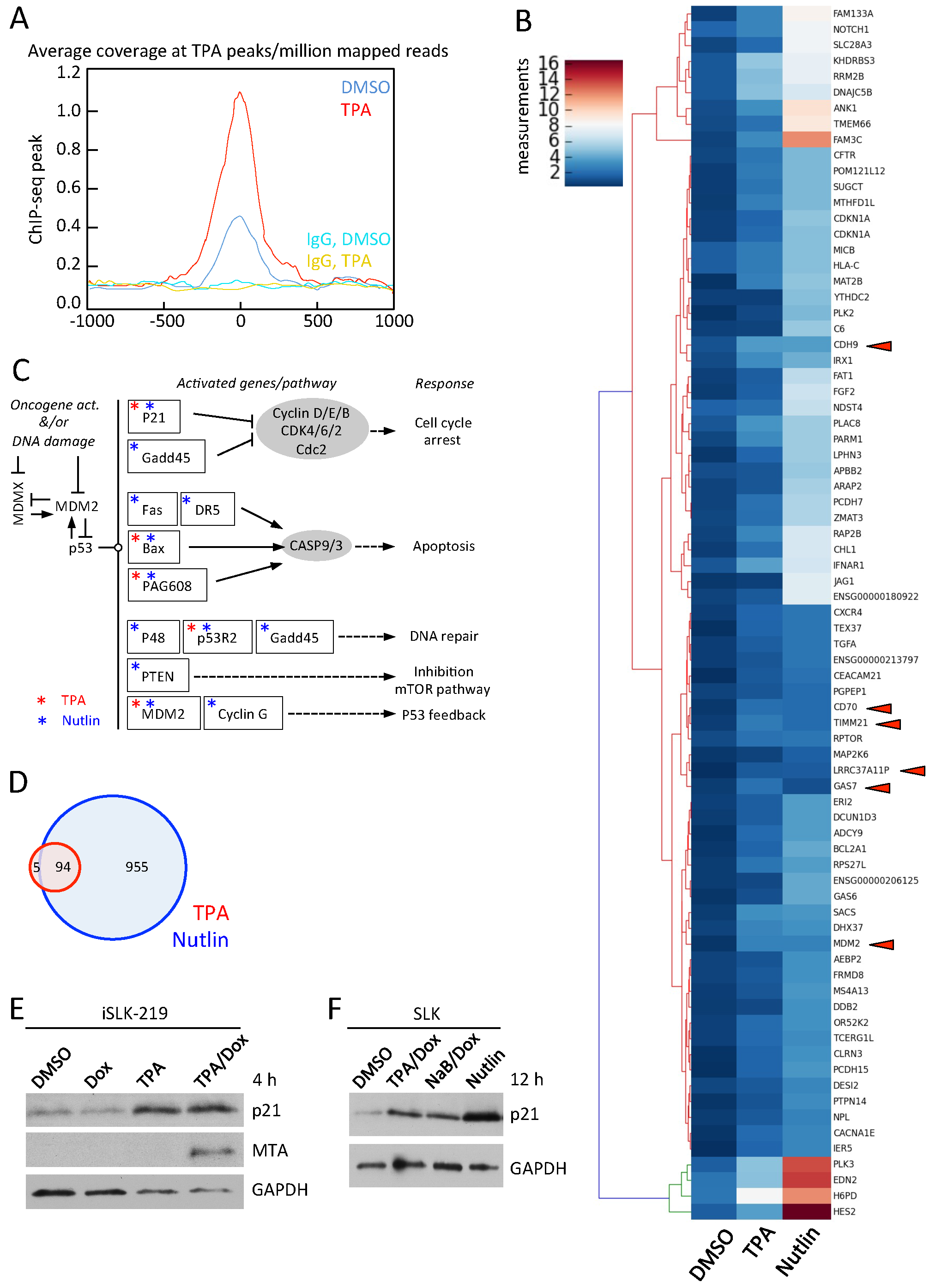

Supplement: S3 Fig — (A) Graphical representation of the average sequencing signal obtained after ChIP-seq of BC-3 cells treated with vehicle (DMSO) or TPA for 24 h. As a negative control, a nonspecific IgG antibody was used. The coverage of ChIP-seq reads extended to the fragment length (with duplicate reads removed) of each sample was calculated for each of the top 99 peaks called from the 24 h sample. The coverage curves were averaged over all peak regions for each sample separately. Finally, the coverage values were normalized to million reads mapped. The graph also includes the background signal from the nonspecific IgG controls (yellow and light blue). (B) Heat-map of the strongest peaks and associated genes identified after ChIP-seq analysis of BC-3 cells treated with DMSO, TPA (24 h) or Nutlin (8 h). The scale has been normalized to reads per peak per million mapped reads. The red arrowheads indicate genes that were associated with similar (or higher) number of reads in cells treated with TPA compared with those obtained from cells treated with Nutlin. (C) Schematic representation of gene-pathways enriched in response to p53 activation after DAVID enrichment analysis. The asterisks indicate the genes identified from the ChIP-seq analysis of TPA (red) or Nutlin (blue) treated cells. Indicated in the scheme are representative genes pooled out from the top 300, statistically significant (p<0.05), peaks in each of the two treatments. (D) The Venn diagrams display the number of peak regions called from the 24 hour TPA sample (N = 99), overlapping with the top 1054 most significant peak-regions (lowest p-values) obtained from Nutlin treated cells. (E-F) iSLK.219 cells (E) or non-infected SLK cells (F) were treated with vehicle (DMSO) or indicated inducers for 4 h (iSLK.219) and 12 h (SLK) and processed for WB using antibodies against p21, GAPDH and also MTA for iSLK.219. (TIF) [file ppat.1005424.s004.tif]

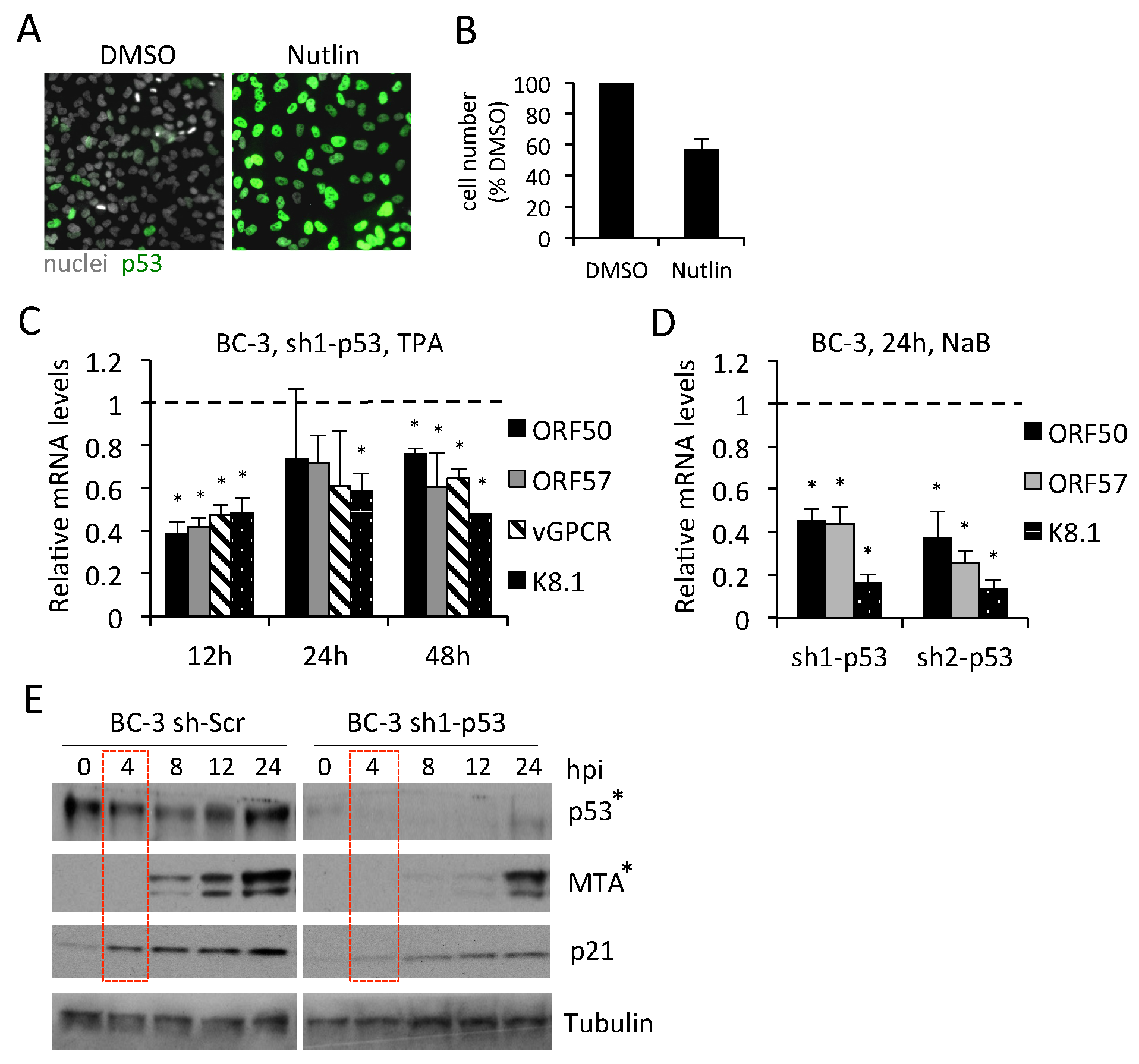

Supplement: S4 Fig — (A) Fluorescence images showing the stabilization of p53 after Nutlin treatment in iSLK.219 treated for 24h and processed for immunofluorescence imaging using antibodies against p53 (green) and hoechst to visualize nuclei (grey). (B) Inhibition of cell growth in iSLK.219 cells treated with Nutlin for 24h. Hoechst-stained nuclei were counted by automated image analysis after high-content fluorescence imaging. Values obtained from Nutlin treated cells were normalized to the cell number obtained in the respective DMSO treated sample. Shown are the average values obtained from three independent experiments. The error bars represent SD. More than 1500 cells were counted in each repetition. (C) mRNA levels of indicated viral lytic genes in BC-3 cells stably expressing sh-Ctrl or sh1-p53 and treated with TPA for indicated times. For each time point, the results are normalized to the values obtained from cells stably expressing the non-specific sh-Ctrl. Error bars represent the SEM. * P<0.05. (D) mRNA levels of indicated viral lytic genes in BC-3 cells stably expressing sh-Ctrl, sh1-p53 or sh2-p53, and treated with NaB for 24 h. For each treatment, the results are normalized to the values obtained from cells stably expressing the non-specific sh-Ctrl and treated with the same inducer. Error bars represent the SEM. * P<0.05. (E) BC-3 cells stably expressing sh-Ctrl or sh1-p53 were treated with TPA for indicated times (hpi). The levels of viral lytic gene expression (MTA), p53 and p21 were analyzed by WB using the respective antibodies. Tubulin was used as a loading control. Note that at 4 hpi, the levels of p21 are much reduced in cells expressing sh1-p53 compared to sh-Ctrl, in the respective time point (red dashed box). (TIF) [file ppat.1005424.s005.tif]

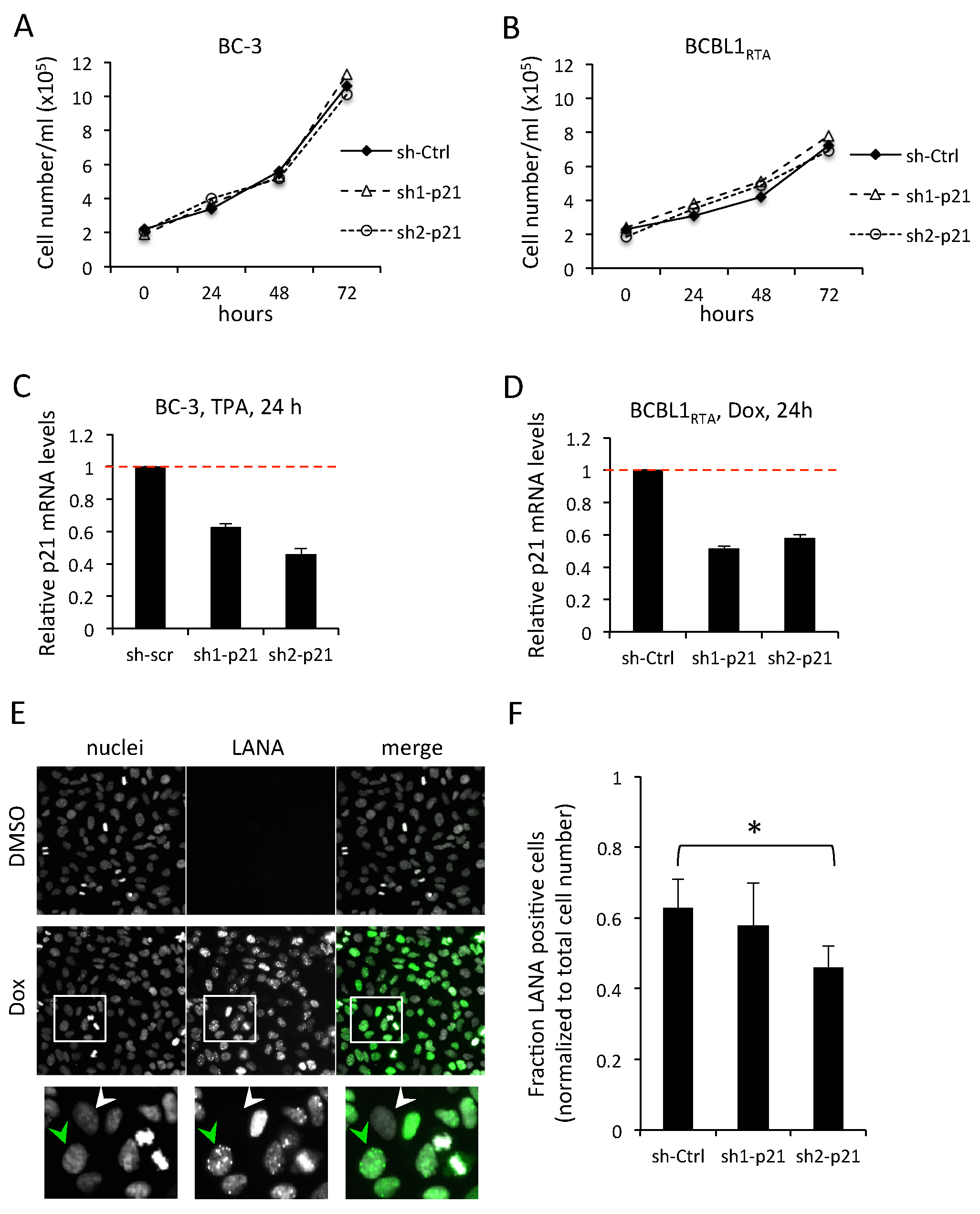

Supplement: S5 Fig — (A-B) Growth curve of BC-3 (A) and BCBL1RTA (B) cells stably expressing indicated shRNA. Cells were counted at indicated time points with a BioRad TC20 automated counter. (C-D) Efficiency of p21 depletion monitored by qRT-PCR in BC3 (C) and BCBL1RTA (D) cells stably expressing sh-Ctrl, sh1-p21 or sh2-p21. Values represent the mean and SDM of three independent experiments and are normalized to sh-Ctrl. (E) Fluorescent images after high-content imaging of U2OS cells infected for 48h with viruses released from the BCBL-1RTA sh-Ctrl cells treated with DMSO or reactivated by Dox, and processed for IF using antibodies against ORF 73 (LANA). The lower panels represent higher magnifications of the respective white-boxed areas. Arrowheads in each image indicate LANA-positive (green arrowhead) of LANA-negative (white arrowhead) cells. (F) Quantification of the fraction of infected (LANA-positive) cells in U2OS cells infected as in (E) with viruses collected from the indicated cell lines. Each value represents the mean and STDEV of three independent experiments. * p<0.05. (TIF) [file ppat.1005424.s006.tif]

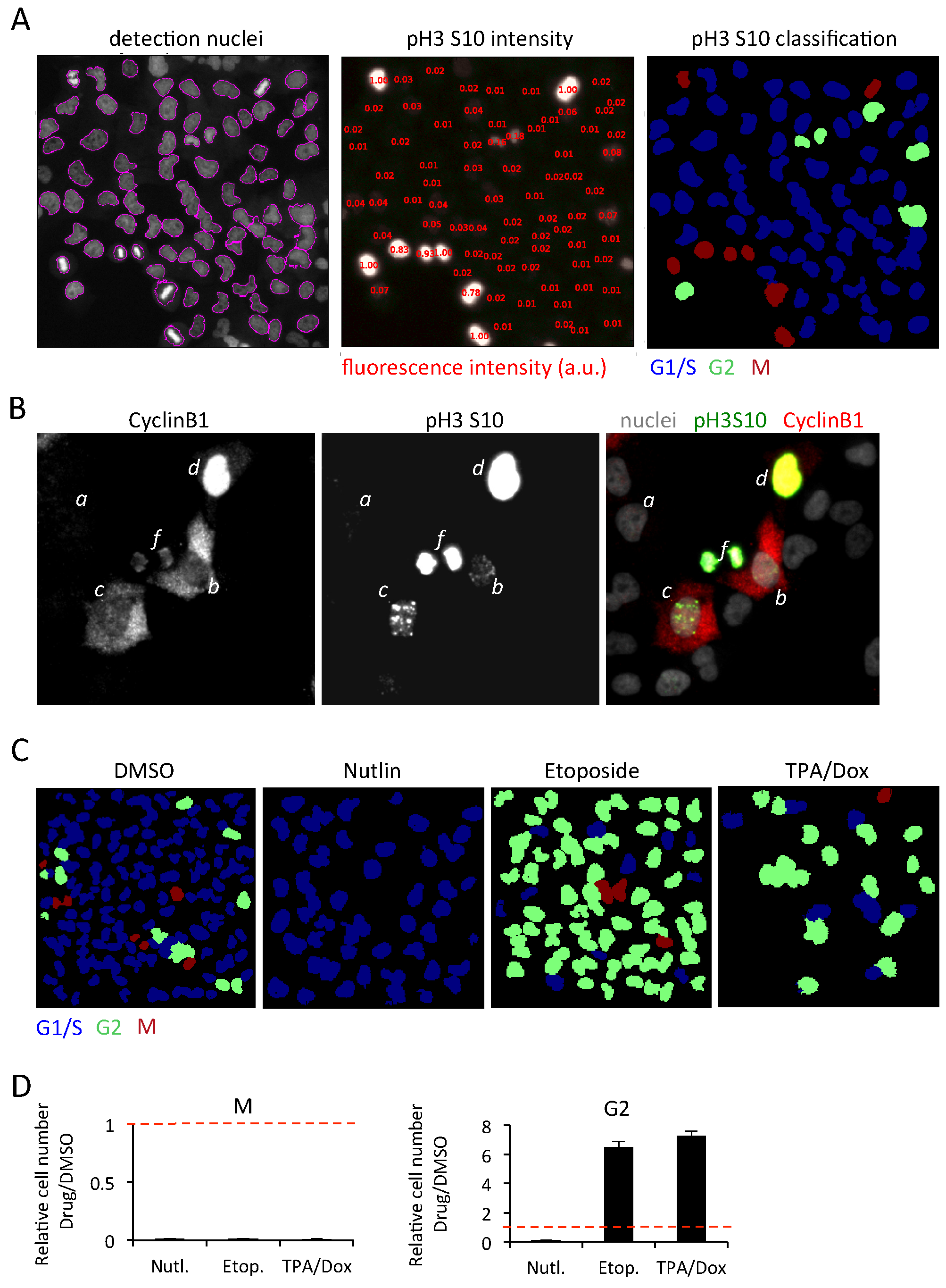

Supplement: S6 Fig — (A) Automated image analysis was performed with the Cell Profiler software to identify an area corresponding to the Hoechst stained nuclei of each cell (purple lines in the left-most image) and, within this area, quantify the intensity of fluorescence of pH3 S10 detected after immunofluorescence staining and imaging (indicated in red in the middle image). Based on the fluorescence intensity of the pH3 S10, cells were classified into G1/S (no signal, blue nuclei), G2 (low intensity, light-green nuclei) and M (bright fluorescence, red nuclei). (B) Fluorescence images of non-induced DMSO-treated iSLK.219 cells after immunofluorescence analysis using antibodies for pH3 S10 (green) and Cyclin B1 (red). The different stages of the cell cycle are indicated by italics letters as in Fig 4A. (C) Digital images after analysis of cell cycle progression with Cell profiler as in A. iSLK.219 cells were treated with either DMSO (0.1%), Nutlin-3A (10 mM, 48h), etoposide (6.25 mM, 24 h) or TPA/Dox for 24 h before pH3 S10 immunostaining, high-content imaging and image analysis. (D) Quantification of cells in M- or G2-phase by image analysis in iSLK.219 cells treated as in C. The values represent the mean and SD of three independent experiments. In each experiment more than 1500 cells were analyzed. For each treatment, values are normalized to the respective DMSO controls (set to one, dashed red line). (TIF) [file ppat.1005424.s007.tif]

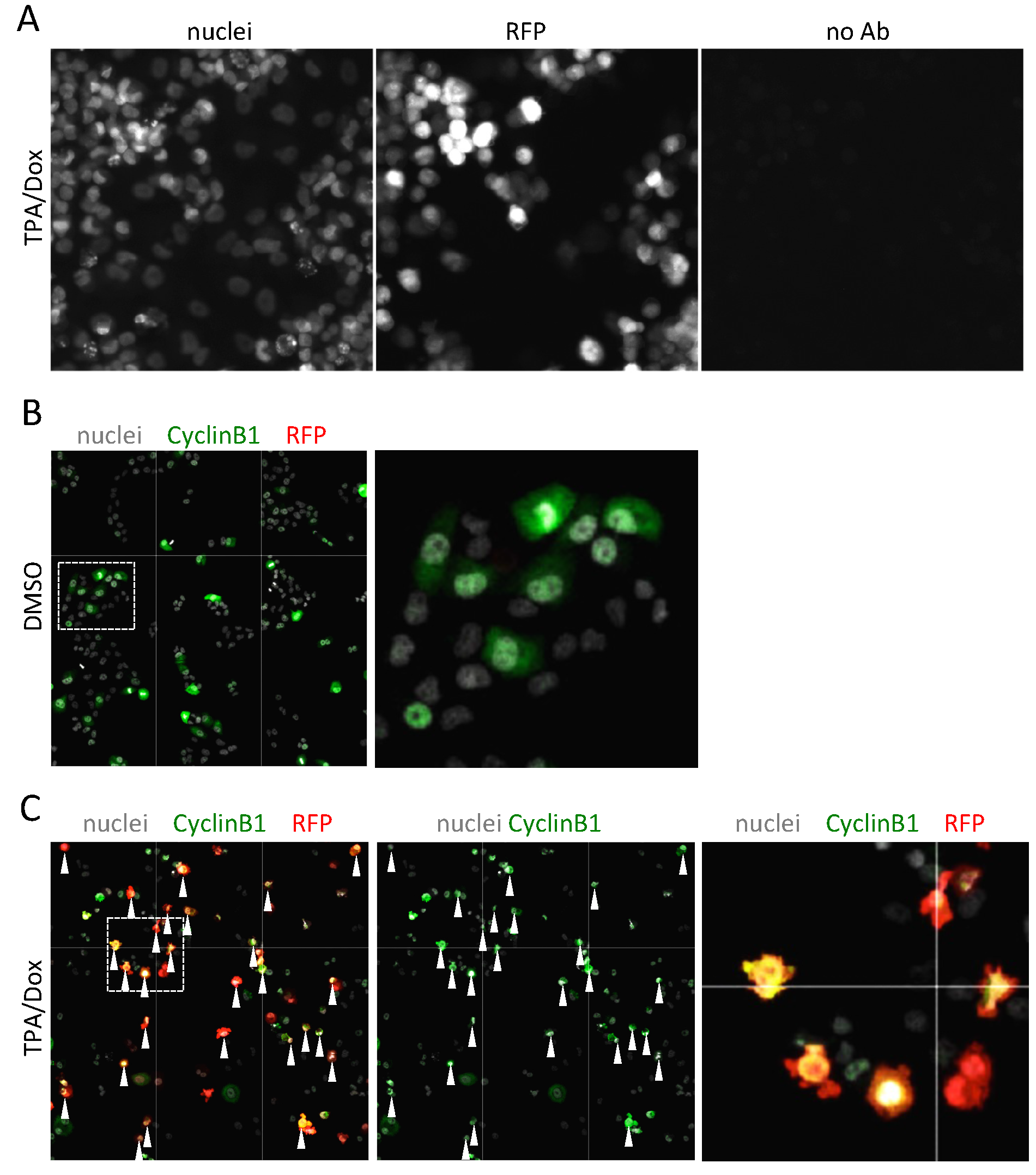

Supplement: S7 Fig — (A) Cross-talk between fluorescent channels. Fluorescence images of iSLK.219 cells treated with TPA/Dox for 20 h and processed for immunofluorescence analysis in the absence of primary antibody and using only the secondary Alexa-647 conjugated antibodies. No fluorescence signal is detected in the far-red channel (no Ab) with the same imaging conditions as in the experiments where the antibody against pH3 S10 was included (compare with Fig 4B). (B-C) Fluorescence images of non-induced (DMSO, C) or induced (TPA/Dox, D) iSLK.219 cells after immunofluorescence analysis using antibodies for Cyclin B1 (green). The RFP expression (red) indicates cells that undergo lytic reactivation. The right-most images are higher magnifications of the white-boxed areas. Arrowheads indicate reactivated cells that are positive for RFP and Cyclin B1. (TIF) [file ppat.1005424.s008.tif]

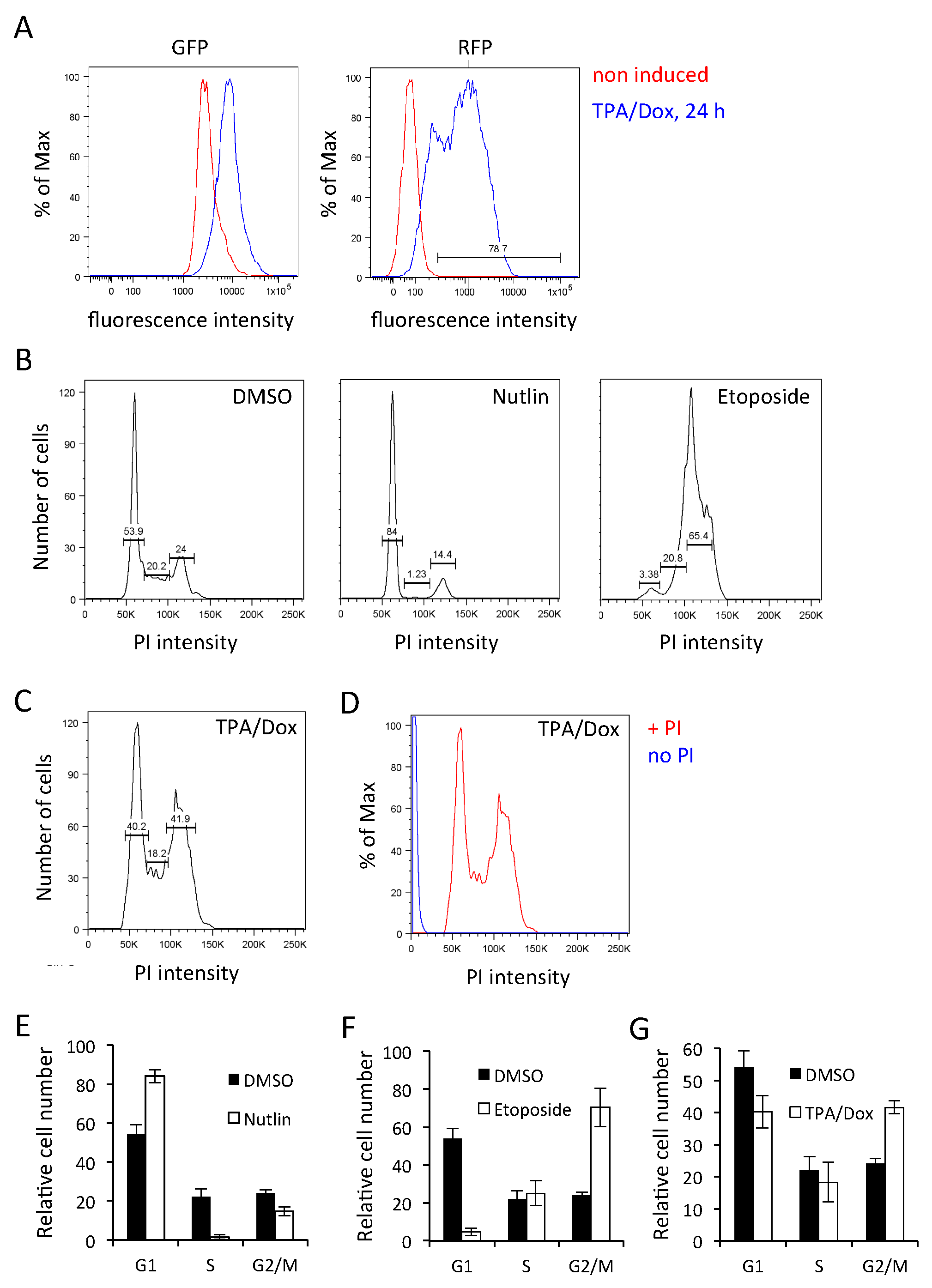

Supplement: S8 Fig — (A) Fluorescence intensity of GFP (left panel) and RFP (right panel) in iSLK.219 cells treated with DMSO (non induced, red line) or TPA/Dox (reactivated, blue line). (B) Cell cycle analysis of iSLK.210 cells treated with indicated drugs for 24 h and stained with PI. (C) Cell cycle analysis of iSLK.210 cells treated with TPA/Dox for 24 h and stained with PI. (D) Comparison of fluorescence signal between TPA/Dox treated iSLK.219 cells stained (red line) or not (blue line) with PI. (E-G) Quantification of cell cycle distribution in iSLK.219 cells treated with the indicated compounds for 24 h and stained with PI. Values represent the mean and SD of three independent experiments. The total number of counted cells in each condition is expressed as 100%. (TIF) [file ppat.1005424.s009.tif]

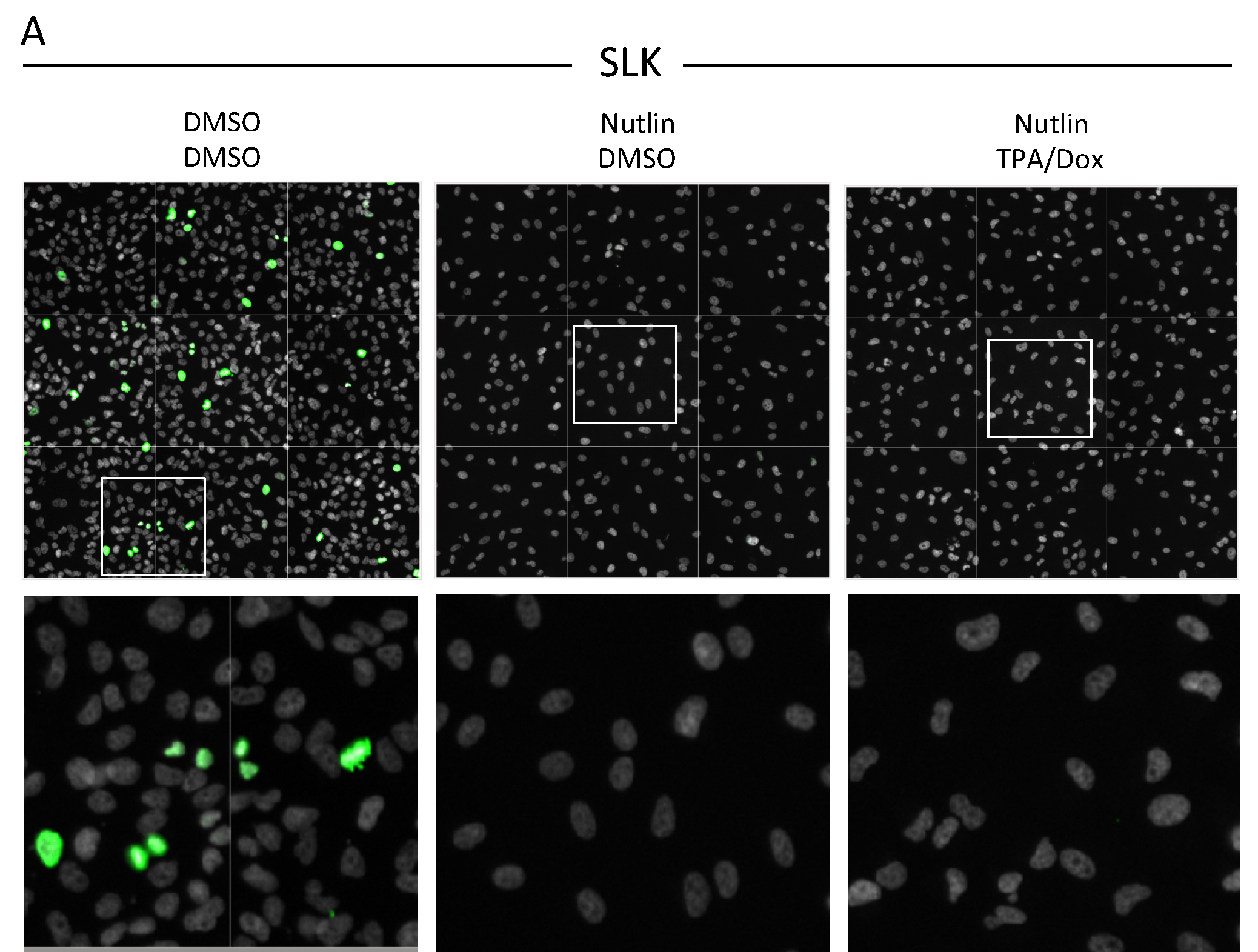

Supplement: S9 Fig — (A) Fluorescent images of SLK cells treated with indicated drugs for 48 h and processed for IF as in Fig 6, using antibodies to detect pH3 S10 (green) and Hoechst to detect nuclei (grey). The lower panels represent higher magnifications of the respective white-boxed areas. Images are the representative of three independent experiments. (TIF) [file ppat.1005424.s010.tif]

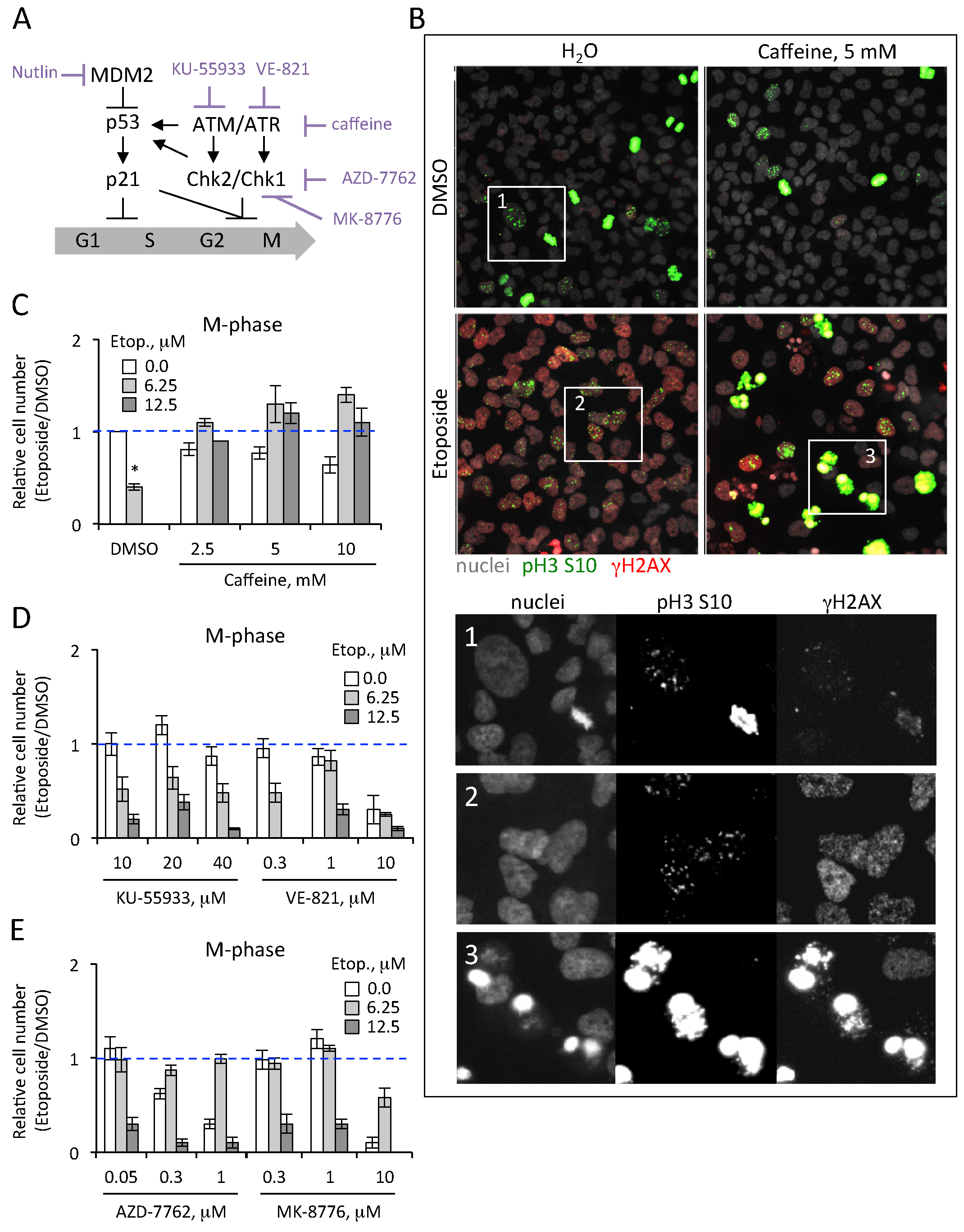

Supplement: S10 Fig — (A) Schematic representation of the DDR and inhibitors (purple) used in this study. (B) Fluorescence images of iSLK.219 cells treated with H2O (control) or caffeine (5 mM) 1 h prior the addition of etoposide (12.5 μM). Cells were fixed 48 h later and processed for immunofluorescence analysis using antibodies against pH3 S10 (green) and γH2AX (red). Nuclei are stained with Hoechst (grey). The lower panels are higher magnifications of the respective white-boxed areas (numbered 1,2 and 3). (C) The ability of Caffeine to inhibit the DDR and restore the cell cycle progression from G2 to M-phase was analyzed by image analysis as described in S5 Fig. iSLK.219 cells were treated with H2O (control) or indicated concentrations of caffeine for 1 h before the incubation with etoposide for 48 hours. For each treatment, values represent the mean and SD of three independent experiments, and are normalized to the number of M-phases detected in H2O treated cells (set as 1, dashed red line). (D-E) The abilities of different inhibitors of DDR to prevent G2-arrest and restore M-phases were tested as in C. For each treatment, values represent the mean and SD of three independent experiments, and are normalized to the number of M-phases detected in DMSO treated cells (set as 1, dashed red line). (TIF) [file ppat.1005424.s011.tif]
